# SMAD4 target genes are part of a transcriptional network that integrates the response to BMP and SHH signaling during early limb bud patterning

**DOI:** 10.1242/dev.200182

**Published:** 2021-12-03

**Authors:** Julie Gamart, Iros Barozzi, Frédéric Laurent, Robert Reinhardt, Laurène Ramos Martins, Thomas Oberholzer, Axel Visel, Rolf Zeller, Aimée Zuniga

**Affiliations:** 1 Developmental Genetics, Department of Biomedicine, University of Basel, 4058 Basel, Switzerland; 2 Functional Genomics Department, Lawrence Berkeley National Laboratory, Berkeley, CA 94720, USA; 3 US Department of Energy Joint Genome Institute, Walnut Creek, CA 94598, USA; 4 School of Natural Sciences, University of California, Merced, CA 95343, USA

**Keywords:** SMAD4, BMP, SHH, Limb development, Anterior, Mouse, ChIP-seq, RNA-seq, Cistrome

## Abstract

SMAD4 regulates gene expression in response to BMP and TGFβ signal transduction, and is required for diverse morphogenetic processes, but its target genes have remained largely elusive. Here, we identify the SMAD4 target genes in mouse limb buds using an epitope-tagged *Smad4* allele for ChIP-seq analysis in combination with transcription profiling. This analysis shows that SMAD4 predominantly mediates BMP signal transduction during early limb bud development. Unexpectedly, the expression of cholesterol biosynthesis enzymes is precociously downregulated and intracellular cholesterol levels are reduced in *Smad4*-deficient limb bud mesenchymal progenitors. Most importantly, our analysis reveals a predominant function of SMAD4 in upregulating target genes in the anterior limb bud mesenchyme. Analysis of differentially expressed genes shared between *Smad4*- and *Shh*-deficient limb buds corroborates this function of SMAD4 and also reveals the repressive effect of SMAD4 on posterior genes that are upregulated in response to SHH signaling. This analysis uncovers opposing trans-regulatory inputs from SHH- and SMAD4-mediated BMP signal transduction on anterior and posterior gene expression during the digit patterning and outgrowth in early limb buds.

## Abstract (precis)

**Summary:** The transcriptional targets of SMAD4 in early limb buds are identified and the largely opposing impact of BMP and SHH signaling on early digit patterning and outgrowth is revealed.

## INTRODUCTION

The transforming growth factor (TGFβ) and bone morphogenetic protein (BMP) pathway constitutes one of the major signaling pathways controlling vertebrate embryonic development (reviewed by Weiss and Attisano, 2013). Of relevance to the present study, BMPs are required for limb bud formation and outgrowth (reviewed by Pignatti et al., 2014). BMP ligands activate their transmembrane BMP receptors (BMPR1A/1B isoforms and BMPR2) that form hetero-tetrameric complexes. The activated BMP receptor complexes trigger R-SMAD (SMAD1, SMAD5 and SMAD8) phosphorylation and form a complex with SMAD4 that translocates to the nucleus and regulates target gene expression together with co-activators or repressors (Weiss and Attisano, 2013). Genetic studies in mice have identified distinct BMP functions during early limb bud development [mouse embryonic day (E) 9.5-E10.0]. High mesenchymal BMP4 activity is required together with BMPR1A and SMAD4-mediated signal transduction in the ectoderm to establish the apical ectodermal ridge (AER) as fibroblast growth factor (FGF) signaling center (Ahn et al., 2001; Bénazet and Zeller, 2013; Pajni-Underwood et al., 2007). Genetic inactivation of *Bmp4* during forelimb bud formation disrupts outgrowth and transcriptional activation of the BMP antagonist gremlin 1 (*Grem1*; Bénazet et al., 2009). In turn, *Grem1* expression progressively lowers mesenchymal BMP activity, which is reinforced by sonic hedgehog (SHH) signaling as part of the self-regulatory SHH/GREM1/AER-FGF feedback signaling system (Bénazet et al., 2009). Genetic analysis in mice has shown that *Shh* is transiently required to specify digit identities in early limb buds and subsequently to promote the proliferative expansion of limb bud mesenchymal progenitors (LMPs; Zhu et al., 2008). Together, these and other studies show that limb bud morphogenesis depends crucially on GREM1-mediated reduction of BMP activity, morphogenetic SHH signaling and proliferation of LMPs as part of the SHH/GREM1/AER-FGF signaling system (Bénazet et al., 2009; Probst et al., 2011). In addition, mesenchymal BMP activity is essential for regulating AER length, which in turn prevents digit polydactyly (Bénazet et al., 2009; Lopez-Rios et al., 2012; Selever et al., 2004). During limb bud outgrowth, SMAD4-mediated signal transduction in the mesenchyme is required together with SHH signaling for positive regulation and propagation of *Grem1* expression. In addition, *Smad4* is required during termination of the self-regulatory SHH/GREM1/AER-FGF signaling system (Scherz et al., 2004; Verheyden and Sun, 2008), as both *Shh* and AER-*Fgf8* expression are prolonged in mouse limb buds lacking mesenchymal *Smad4* (Bénazet et al., 2012). Finally, SMAD4-mediated BMP signal transduction is required to initiate the aggregation and differentiation of the chondrogenic progenitors of the limb skeletal primordia (Bénazet et al., 2012; Lopez-Rios et al., 2012; Pizette and Niswander, 2000). In contrast, there are no genetic data pointing to essential functions of TGFβ signaling during early limb bud development, but a study using cultured limb bud cells provided evidence that TGFβ signaling alleviates an inhibitory effect of BMPs on specification of *Sox9*-positive osteochondrogenic progenitors (Karamboulas et al., 2010).

To gain an unbiased view of how SMAD4 mediates signal transduction during early mouse forelimb bud development, we have identified its direct transcriptional targets (i.e. SMAD4 target genes) using a novel *Smad4* allele with an inserted 3xFLAG epitope tag (*Smad4*^3xF^ allele). This *Smad4*^3xF^ allele allows sensitive and unbiased detection of the genomic regions enriched in endogenous SMAD4-chromatin complexes using ChIP-seq analysis. Combining the SMAD4 cistrome with RNA-seq analysis of wild-type and *Smad4*^Δ/Δc^ mouse forelimb buds that lack mesenchymal *Smad4* identifies the SMAD4 target genes among the differentially expressed genes (DEGs) in early limb buds. This analysis reveals an unexpected SMAD4 requirement for maintaining the expression of cholesterol biosynthesis enzymes in early limb buds because, in *Smad4*^Δ/Δc^ forelimb buds, their expression is prematurely downregulated and endogenous cholesterol levels are reduced in mutant LMPs. We also identify the direct SMAD4 targets in the TGFβ and BMP pathways, which establishes that the *Smad4* deficiency preferentially disrupts BMP signal transduction in early forelimb buds. Whole-mount *in situ* hybridization screening identifies SMAD4 target genes whose spatial expression is altered in early limb buds. Furthermore, *LacZ* reporter analysis shows that the SMAD4-interacting enhancers for some target genes are active in the anterior forelimb bud mesenchyme. Together with the observed spatial changes in gene expression, this points to SMAD4 functions in upregulating target gene expression in the anterior forelimb bud mesenchyme. Comparative analysis of DEGs in *Smad4*^Δ/Δc^ and *Shh*^Δ/Δc^ forelimb buds (Probst et al., 2011) identifies the genes co-regulated by both pathways in early limb buds. Gene regulatory network (GRN) analysis reveals the interactions of SMAD4-mediated BMP signal transduction with SHH signaling in the spatial regulation of key genes during the early phase of digit specification (Zhu et al., 2008) and establishment of the self-regulatory signaling system (Bénazet et al., 2009).

## RESULTS

### Identification of the SMAD4 cistrome and target genes in mouse forelimb buds

Specific detection of the endogenous SMAD4 protein complexes was achieved by inserting a 3xFLAG (3xF) epitope tag into the SMAD4 C-terminal domain using homologous recombination in mouse ES cells (Fig. S1). Homozygous *Smad4*^3xF/3xF^ mice are born at the expected Mendelian ratios and display no overt phenotypes. No alterations of limb bud development were detected in *Smad4*^3xF/3xF^ embryos, which contrasts with the disrupted chondrogenesis and skeletal development in mouse limb buds lacking mesenchymal *Smad4* (Bénazet et al., 2012). In early mouse forelimb buds, the distribution of SMAD4^3xF^ proteins is uniform. Higher levels of SMAD4 proteins are detected in the cytoplasm than in the nucleus, but diffuse nucleoplasmic staining is detected in most mesenchymal cells of *Smad4*^3xF/3xF^ forelimb buds at embryonic day E9.5 (Fig. S1, see also Bénazet et al., 2012).

**Fig. 1.**
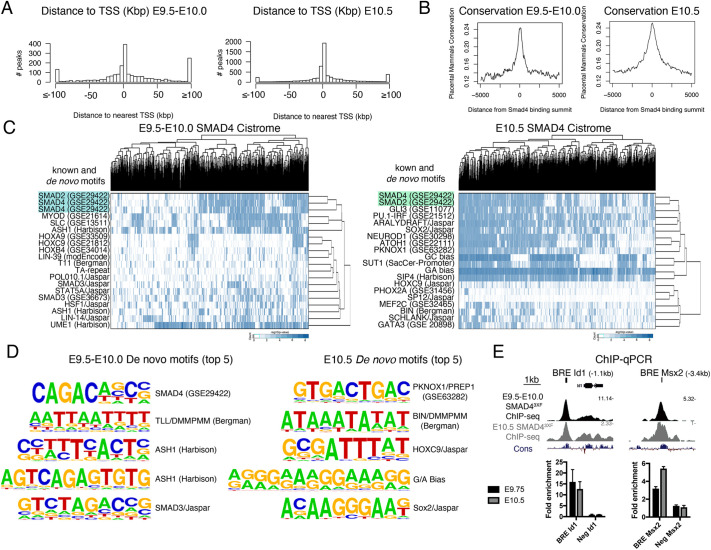
**Identification of the genomic regions enriched in SMAD4-chromatin complexes in mouse forelimb buds.** (A) Histogram showing the distribution of SMAD4-interacting regions in relation to the nearest transcriptional start site (TSS) at E9.5-E10.0 (25-30 somites) and E10.5 (34-38 somites). (B) The average *Phastcons* conservation of the genomic regions enriched in SMAD4-chromatin complexes is shown at both stages. (C) Hierarchical clustering of the high-affinity matches for the top known and *de novo* motifs enriched in the SMAD4-bound regions at both stages. (D) The top five *de novo* motif identified in the genomic regions enriched in SMAD4-chromatin complexes in forelimb buds at both stages. (E) ChIP-qPCR validation of two previously known SMAD4-interacting genomic regions – BREs for *Id1* and *Msx2*, respectively – at both stages. Two biological replicates were analyzed (data are mean±s.d. of three technical replicates).

Forelimb buds of *Smad4*^3xF/3xF^ embryos at two stages were used to identify the SMAD4 cistromes during the onset of limb bud development with high mesenchymal BMP activity (E9.5-E10.0, 25-30 somites) and during early outgrowth, when BMP signal transduction is lowered by GREM1-mediated BMP antagonism (E10.5, 34-38 somites, Bénazet et al., 2009). The SMAD4 cistrome of forelimb buds at both stages was determined using chromatin immunoprecipitation in combination with next-generation sequencing (ChIP-seq, Fig. 1). Two biological replicates consisting each of ∼80 dissected forelimb buds were analyzed per stage. The dissected forelimb buds included some proximal trunk tissue to also detect interactions with genes expressed early in the proximal limb bud and flank mesenchyme. For early limb buds (E9.5-E10.0), statistical analysis of the two replicates by MACS and MSPC identified 2073 significantly and reproducibly enriched SMAD4 ChIP-seq peaks (Jalili et al., 2015). About 40% of them are located close to transcriptional start sites (TSS, ±5 kb) while ∼20% are located ≥100 kb away from TSS (left panel, Fig. 1A). During limb bud outgrowth (E10.5), 6185 significantly enriched and conserved SMAD4 ChIP-seq peaks were identified, most of which are also located close to TSS (right panel, Fig. 1A). Evolutionary conservation analysis shows that the peak summits of the genomic regions enriched in SMAD4-chromatin complexes are more conserved than the flanking regions in placental mammals (Fig. 1B). Enrichment analyses for known and *de novo* motifs using HOMER identified the SMAD consensus binding motifs as the most enriched motifs at both stages (Fig. 1C,D; Heinz et al., 2010). In addition, the PKNOX1/PREP1 homeobox motif is the most enriched *de novo* motif in the SMAD4^3xF^ forelimb bud cistrome at E10.5 (right panel, Fig. 1D). This could be functionally relevant as the TALE homeodomain transcription factors PKNOX1/PREP1 and PBX1 interact with SMAD4 to regulate gene expression in cultured cells (Bailey et al., 2004). However, our analysis also revealed significant differences in the overall binding motifs enriched in SMAD4-chromatin complexes from forelimb buds characterized by high (E9.5-E10.0) and low (E10.5) mesenchymal BMP activity (Fig. 1C,D). Furthermore, the BMP responsive elements (BREs; Brugger et al., 2004; Korchynskyi and ten Dijke, 2002) located near the *Id1* and *Msx2* genes are significantly enriched in the SMAD4^3xF^ ChIP-seq datasets at both stages, which was confirmed by ChIP-qPCR analysis (Fig. 1E). Therefore, the two SMAD4^3xF^ cistromes constitute valid resources to identify the limb bud mesenchymal SMAD4 target genes during the onset (E9.5-E10.0) and distal progression (E10.5) of forelimb bud development.

### Identification of differentially expressed SMAD4 target genes

**Fig. 2.**
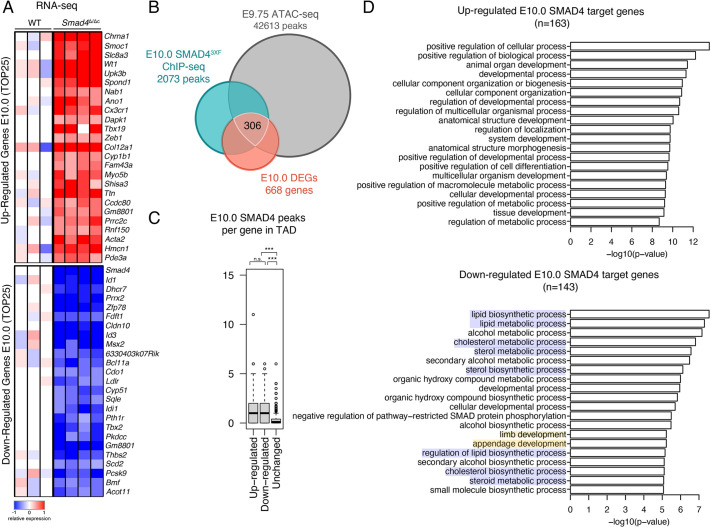
**Identification of SMAD4 target genes in mouse forelimb buds at E10.0.** (A) Heatmaps showing the DEGs identified by comparing wild-type (WT) and *Smad4*^Δ/Δc^ transcriptomes at E10.0 (30 somites; wild type, *n*=3; *Smad4*^Δ/Δc^, *n*=4 biological replicates). The top 25 up- and downregulated genes are shown. For each gene, the log2-ratio between the expression level in each sample and the mean of the three biological replicates for the wild-type forelimb buds is shown. Red indicates increased expression and blue indicates reduced expression in comparison with the mean of the wild-type samples. (B) Three-way Venn diagram showing the intersection between the ChIP-seq (E9.5-10.5, 25-30 somites), ATAC-seq (E9.75, 26 somites) and RNA-seq (E10.0, 30 somites) datasets. This identifies 306 candidate SMAD4 target genes in mouse forelimb buds. (C) Box plot representing the number of E9.5-E10.0 SMAD4^3xF^ ChIP-seq peaks within a TAD of genes that are either up- or downregulated in *Smad4*^Δ*/*Δ*c*^ forelimb buds in comparison with genes with unaltered expression. The box plot indicates median (50th percentile) and interquartile range (25th and 75th percentiles; IQR) with whiskers set at 1.5×IQR. Values exceeding the whiskers are considered outliers and are individually marked. Upregulated versus unchanged, *P*=5.2e-26; downregulated versus unchanged, *P*=9.9e-32 (Mann–Whitney test). n.s., not significant. (D) GO enrichment analysis of biological processes of the up- and downregulated SMAD4 target genes in *Smad4*^Δ*/*Δ*c*^ forelimb buds. The GO terms for processes relevant to sterol/lipid/sterol/cholesterol biosynthesis and to limb development are highlighted in blue and yellow, respectively (bottom panel).

**Fig. 3.**
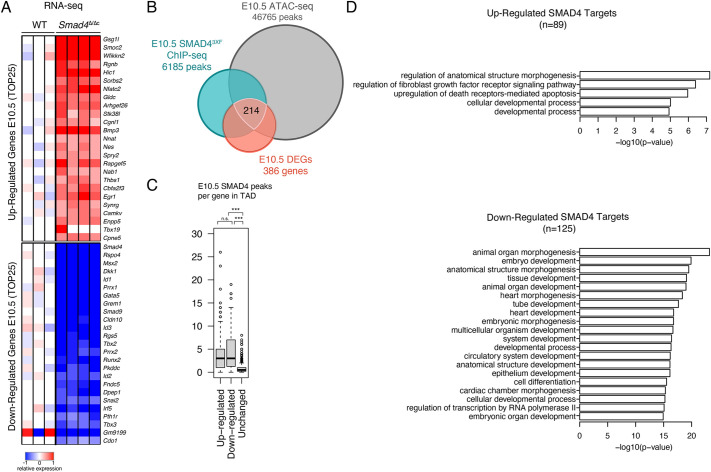
**The SMAD4 target genes in mouse forelimb buds at E10.5.** (A) Heatmaps showing the DEGs identified by comparing wild-type (WT) and *Smad4*^Δ/Δc^ transcriptomes at E10.5 (35 somites; wild type, *n*=3; *Smad4*^Δ/Δc^, *n*=4 biological replicates). The top 25 downregulated and upregulated genes are shown (normalized to the mean expression in wild-type samples). For each gene, the log2-ratio between the expression levels in each sample and the mean of the wild-type forelimb buds is shown. (B) Three-way Venn diagram showing the intersection between the ChIP-seq (E10.5, 34-38 somites), ATAC-seq (E10.5, 35 somites) and RNA-seq (E10.5, 35 somites) datasets. This intersection identifies 214 candidate SMAD4 target genes in forelimb buds. (C) Box plot analysis representing the number of SMAD4^3xF^ ChIP-seq peaks within a TAD harboring the SMAD4 target gene. The box plot indicates median (50th percentile) and interquartile range (25th and 75th percentiles; IQR) with whiskers set at 1.5×IQR. Values exceeding the whiskers are considered outliers and are individually marked. Upregulated versus unchanged, *P*=1.5e-52; downregulated versus unchanged, *P*=1.4e-36 (Mann–Whitney test). n.s., not significant. (D) GO enrichment analysis of biological processes for down- and upregulated SMAD4 target genes.

RNA-seq was used to identify the DEGs in the mesenchyme of wild-type and *Smad4*-deficient forelimb buds (Figs 2 and 3). As *Smad4*-deficient mouse embryos die before the onset of limb bud development, *Smad4* was conditionally inactivated in the forelimb bud mesenchyme using the *Prrx1*-CRE transgene (*Smad4*^Δ/Δc^). *Prrx1*-CRE-mediated *Smad4* inactivation results in clearance of mesenchymal SMAD4 proteins by around E10.0. Owing to disruption of chondrogenesis, this results in complete loss of limb skeletal elements, but in the early forelimb bud stages analyzed no mesenchymal apoptosis or other morphological abnormalities are observed (Bénazet et al., 2012). First, pairs of age-matched wild-type and *Smad4*^Δ/Δc^ forelimb buds were analyzed at E10.0 (30 somites) because at this stage the SHH/GREM1/AER-FGF feedback signaling system is being established (Fig. 2A and Tables S1 and S2; Bénazet et al., 2009). Comparison of wild-type and *Smad4*^Δ/Δc^ forelimb buds identified 668 DEGs (Fig. 2B; fold change ≥1.2; FDR ≤0.1). The cut-off was set at ≥1.2 to allow detection of spatial differences by whole-mount RNA *in situ* hybridization (Probst et al., 2011). Among the 668 DEGs in early *Smad4*^Δ/Δc^ forelimb buds, 360 are upregulated and 308 are downregulated (Tables S1 and S2).

The SMAD4 transcriptional target genes in early mouse forelimb buds (Fig. 2B) were identified as follows: the SMAD4 ChIP-seq peaks (E9.5-E10.0, 25-30 somites) overlapping regions of open chromatin, as determined by ATAC-seq analysis in wild-type forelimb buds at E9.75 (26 somites, *n*=2), were assigned to the nearest DEG (E10.0, 30 somites) that is located within a maximally 1Mb genomic interval, which corresponds to the average size of topologically associating domains (TADs, Dixon et al., 2016, 2012). This bioinformatics analysis identified 306 SMAD4 target genes that are differentially expressed in the mesenchyme of early mouse forelimb buds. Genes that are either up- or downregulated in *Smad4*^Δ*/*Δ*c*^ forelimb buds contain, in general, more SMAD4-binding regions within their TADs than genes whose expression is unchanged (Fig. 2C). In *Smad4*^Δ/Δc^ forelimb buds at E10.0, the expression of 163 SMAD4 target genes is upregulated (Table S3), whereas the others are downregulated (*n*=143, Table S4). Gene ontology (GO) analysis shows that genes with increased expression, i.e. target genes negatively regulated by SMAD4, function in various developmental processes, in agreement with the broad *Smad4* requirement during embryonic development (upper panel, Fig. 2D; Chu et al., 2004). In addition to its functions in limb development (yellow-shaded terms, lower panel, Fig. 2D), GO analysis of SMAD4 target genes with reduced expression in mutant forelimb buds reveals an unexpected role for SMAD4 in the positive regulation of lipid/sterol/cholesterol biosynthesis and metabolism (blue-shaded terms, Fig. 2D).

As BMP activity is progressively reduced due to increasing GREM1-mediated BMP antagonism (Bénazet et al., 2009), we used the same strategy to identify DEGs and SMAD4 target genes in forelimb buds at E10.5 (35 somites, Fig. 3 and Tables S5-S8). Comparison of wild-type and *Smad4*^Δ/Δc^ forelimb buds identified 386 DEGs and 214 differentially expressed SMAD4 target genes (Fig. 3A,B). As for the earlier limb bud stage, more SMAD4-binding regions were detected in TADs encoding DEGs than in TADs containing genes with unchanged expression in *Smad4*^Δ/Δc^ limb buds (Fig. 3C). This indicates that target genes whose limb bud expression depends crucially on SMAD4 are regulated by the interaction of SMAD4-chromatin complexes with multiple rather than single *cis*-regulatory modules (CRMs, Fig. 3C). GO analysis of the SMAD4 target genes in forelimb buds at E10.5 points to functions in various developmental processes (Fig. 3D), but terms relevant to lipid/sterol/cholesterol biosynthesis are no longer enriched (compare to Fig. 2D). This indicates that SMAD4 upregulates the expression of enzymes involved in cholesterol synthesis during the onset rather than during the progression of forelimb bud development.

### Premature transcriptional downregulation of cholesterol synthesis enzymes and intracellular cholesterol in *Smad4*-mutant limb buds

**Fig. 4.**
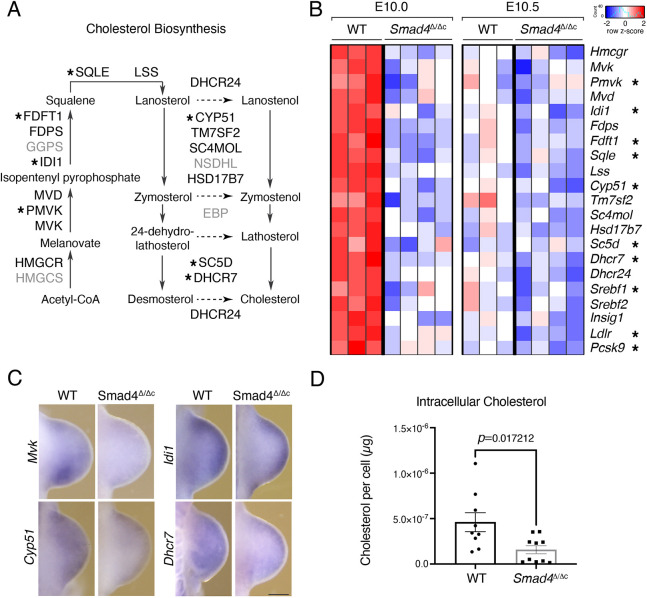
**The expression of cholesterol biosynthesis enzymes is prematurely downregulated in *Smad4*^Δ/Δc^ limb buds.** (A) Schematic representation of the cholesterol biosynthesis pathway. Enzymes downregulated in *Smad4*^Δ/Δc^ forelimb buds at E10.0 are indicated in black, direct SMAD4 target genes are indicated by asterisks. Enzymes with unchanged expression are indicated in gray. (B) Heatmap showing the expression of the downregulated genes encoding enzymes and regulators of cholesterol biosynthesis in wild-type (WT) and *Smad4*^Δ/Δc^ forelimb buds at E10.0 (30 somites) and E10.5 (35 somites). For each gene, the z-score of the expression levels is shown. Red indicates higher and blue indicates lower expression compared with the average expression level. SMAD4 target genes are indicated with asterisks. (C) Whole-mount *in situ* hybridization analysis of key genes in the cholesterol biosynthesis pathway, the spatial expression of which is clearly altered in *Smad4*^Δ/Δc^ forelimb buds at E10.0 (28-31 somites). For each gene and genotype, *n*=3 biological replicates from three independent experiments were analyzed. Scale bar: 250 µm. (D) Biochemical quantitation of intracellular cholesterol in wild-type (*n*=9) and *Smad4*^Δ/Δc^ (*n*=10 biological replicates) LMPs after culture in cholesterol-free medium (20-24 h). (*P*=0.017212, Mann–Whitney test). Data are mean±s.e.m. with individual data points shown.

The transcript levels of cholesterol biosynthesis enzymes (reviewed by Luo et al., 2020) are higher in wild-type than *Smad4*^Δ/Δc^ forelimb buds at E10.0 (Fig. 4A,B, Tables S2, S6). Only by E10.5 are expression levels reduced to a similar extent in both genotypes, which reveals the SMAD4 requirement for upregulating/maintaining the transcription of cholesterol biosynthesis enzymes during the onset of limb bud development (prior to E10.5, Fig. 4B). Several of these downregulated enzymes are direct transcriptional targets of SMAD4 at E10.0 (7 of 16, Fig. 4A,B and Table S4). The target genes that are prematurely downregulated in *Smad4*^Δ/Δc^ forelimb buds also include non-enzymatic regulators of the cholesterol pathway, such as *Insig1, Ldlr*, *Pcsk9* and *Srebf1* (Fig. 4B; Luo et al., 2020). Comparative whole-mount RNA *in situ* hybridization (WISH) analysis shows that most cholesterol biosynthesis enzymes and regulators are expressed rather uniformly, which precludes detection of distinct spatial differences (Fig. S2). However, the spatial transcript distribution of key enzymes such as *Mvk, Idi1*, *Cyp51* and *Dhcr7* is altered in *Smad4*^Δ/Δc^ forelimb buds (Fig. 4C). Together with reduced transcript levels for most enzymes (panel E10.0 in Fig. 4B), this points to possible alterations in endogenous cholesterol biosynthesis in mutant limb buds at early stages (E10.0). The total cholesterol content includes both cell membrane-associated and intracellular cholesterol, and a potential deficiency in endogenous cholesterol could be masked by exogenous cholesterol, produced by other embryonic tissues or of maternal origin (Tint et al., 2006). Therefore, the levels of intracellular cholesterol were analyzed as follows: wild-type and *Smad4*^Δ/Δc^ LMPs were isolated from pairs of forelimb buds (E10.0; 28-30 somites) and cultured in cholesterol-free medium for 20-24 h. After depletion of membrane-associated cholesterol, intracellular cholesterol levels were quantitated for LMPs of both genotypes (Table S9; Vienken et al., 2017; Wilhelm et al., 2017) and the intracellular cholesterol levels per cell determined (Fig. 4D). Although there is inherent variability between LMPs from different forelimb buds, wild-type LMPs contain, on average, ∼4.60×10^−7^ µg intracellular cholesterol, whereas these levels are reduced to ∼1.47×10^−7^ µg cholesterol per cell in *Smad4*^Δ/Δc^ limb buds (Fig. 4D). This intracellular cholesterol deficiency is a likely consequence of the premature downregulation of cholesterol biosynthesis enzymes in mutant forelimb buds (E10.0 in Fig. 4B). As cholesterol modification is required for SHH signaling (Li et al., 2006), we investigated potential alterations in cultured *Smad4*^Δ/Δc^ LMPs. However, cellular and biochemical analysis failed to reveal significant alterations, possibly because endogenous cholesterol synthesis is reduced by the *Smad4* deficiency rather than disrupted by, for example, inactivating the *Dhcr7* or the *Sc5d* enzymes (Fig. 4A; Cooper et al., 2003; Krakowiak et al., 2003). In agreement, no significant spatial changes in *Shh* expression and its targets *Gli1* and *Ptch1*, which serve as transcriptional sensors of SHH signal transduction, are detected in *Smad4*^Δ/Δc^ forelimb buds (Fig. S3). In contrast, the SMAD4 target gene *Hhip*, which encodes an inhibitor of SHH signaling (Chuang and McMahon, 1999), is upregulated in *Smad4*^Δ/Δc^ forelimb buds at E10.5 (Fig. S3, Tables S5 and S7).

### The *Smad4* deficiency disrupts BMP signal transduction during early forelimb bud development

**Fig. 5.**
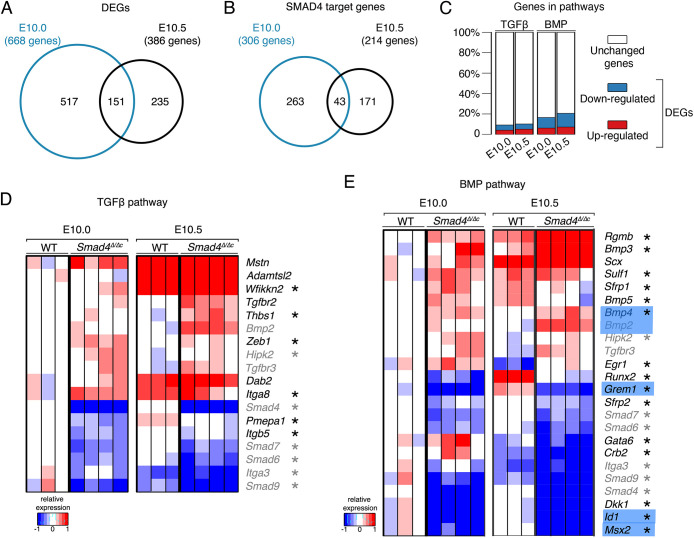
**SMAD4 differentially regulates gene expression in forelimb buds, but predominantly impacts the BMP pathway at E10.0 and E10.5.** (A,B) Venn diagrams showing the intersection between the DEGs at E10.0 and E10.5 (A), and SMAD4 target genes at E10.0 and E10.5 (B). (C) Stacked bar plots show the percentage of DEGs functionally associated with the TGFβ (GO:0007179) and BMP (GO:0030509) pathways, respectively. (D,E) Heat maps showing the DEGs in the TGFβ (D) and BMP (E) pathways. For each gene, the log2-ratio between the expression level in each sample (E10.5 in wild-type, E10.0 and E10.5 *Smad4*^Δ/Δc^) and the mean of the wild-type forelimb buds at E10.0 are shown. Red indicates increased expression and blue indicates reduced expression in comparison with the mean of the wild-type samples. Genes indicated in black are either TGFβ or BMP pathway specific; genes indicated in gray are shared between the two pathways. The names of some key genes in the BMP pathway are highlighted in blue. Asterisks mark the SMAD4 target genes among the DEGs.

To gain insight into major molecular differences between wild-type and *Smad4*-deficient limb buds, the stage-specific and shared DEGs and SMAD4 target genes were identified (Fig. 5A,B). Not only is the number of DEGs and SMAD4 target genes reduced in forelimb buds at E10.5, but also few DEGs and SMAD4 target genes are shared between the two stages (DEGs, *n*=151; targets, *n*=43, Fig. 5A,B, Tables S10-S13). Rather, most of the DEGs and SMAD4 target genes are markedly different during the onset (E9.5-E10.0) and progression of forelimb bud development (E10.5; Figs 2, 3 and 5A,B). Interestingly, this change in SMAD4 target genes correlates well with the observed differences in the enriched motifs in SMAD4-chromatin complexes (Fig. 1D) and parallels the shift from high to low BMP activity (Bénazet et al., 2009). As SMAD4 participates in both BMP and TGFβ signal transduction, we assessed the extent to which the expression of DEGs and inferred SMAD4 target genes (Figs 2 and 3) in the TGFβ and BMP pathways are altered between wild-type and *Smad4*^Δ/Δc^ forelimb buds (TGFβ GO:0007179 and BMP GO:0030509; Fig. 5C-E). This analysis shows that a smaller fraction of genes assigned to the TGFβ than BMP pathway are differentially expressed (Fig. 5C). Only six of the 17 DEGs belonging to the TGFβ pathway are downregulated in mutant forelimb buds, whereas others are precociously upregulated (Fig. 5C,D). In contrast, analysis of the BMP pathway shows that the fraction of downregulated DEGs increases during early limb bud outgrowth (from 11 to 15 of the 23 DEGs, Fig. 5C,E). This is intriguing as it parallels the reduction in BMP activity during progression of wild-type limb bud outgrowth, which does not occur in *Smad4*^Δ/Δc^ forelimb buds (Fig. 5E; Bénazet et al., 2009, 2012). Furthermore, the *Bmp2*, *Bmp4* and *Bmp7* ligands, which are required in the limb bud mesenchyme, are up-regulated, whereas the expression of transcriptional sensors for BMP signal transduction, *Msx2* and *Id1*, is much reduced in *Smad4*^Δ/Δc^ forelimb buds (Fig. 5E, Fig. S4; Brugger et al., 2004; Lopez-Rovira et al., 2002). As no corresponding changes are detected in the *Tgfb* pathway (Fig. 5D, Fig. S4), *Smad4* functions predominantly in BMP signal transduction during early limb bud development (E10.0-E10.5). The opposing effects of mesenchymal *Smad4* deficiency on the expression of *Bmp* ligands and transcriptional sensors shows that BMP signal transduction is disrupted in the *Smad4*-mutant mesenchyme. This is corroborated by the failure to upregulate mesenchymal *Grem1* via the feedback signaling system in response to increased *Bmp4* expression (Fig. 5E; Bénazet et al., 2009).

### *Smad4* controls the spatial upregulation of target genes by interacting with enhancers active in the anterior forelimb bud mesenchyme

**Fig. 6.**
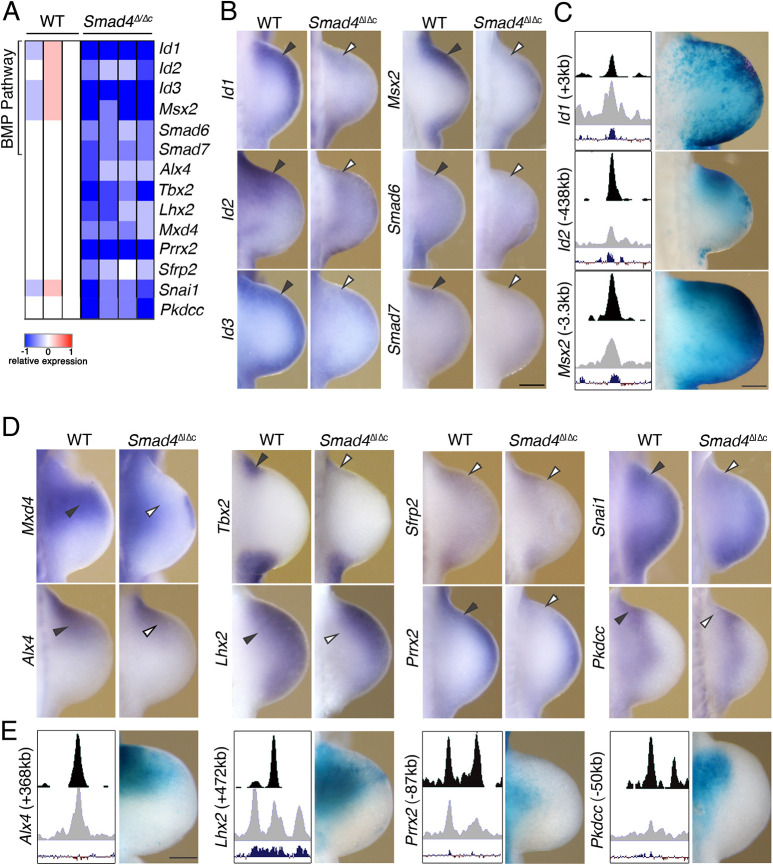
**Target genes positively regulated by SMAD4 in the anterior forelimb bud mesenchyme.** (A) Heat map of the target genes, the expression of which is positively regulated by SMAD4 in the anterior forelimb bud mesenchyme. For each gene, the log2-ratio between the expression level in each sample and the mean of the three biological replicates for wild-type (WT) forelimb buds is shown. Red indicates increased expression and blue indicates reduced expression in comparison with the mean of the wild-type samples. (B,D) Comparative whole-mount *in situ* hybridization analysis of selected BMP pathway genes (B) and SMAD4 target genes (D) whose spatial expression in the anterior limb bud mesenchyme is altered in *Smad4*^Δ/Δc^ forelimbs at E10.0 (28-31 somites). (C,E) Analysis of the *LacZ* reporter activity of SMAD4-enriched candidate CRMs associated with selected target genes. Left panels show a scheme depicting the genomic region harboring the CRM with the SMAD4 ChIP-seq peak (top), the ATAC-seq peak (middle) and the evolutionary conservation (bottom). Right panels show the *LacZ* reporter activity of SMAD4-enriched candidate CRMs in independent transgenic founders embryos with forelimb bud mesenchymal expression at E10.5 for *Id1* (*n*=4), *Id2* (*n*=6), *Msx2* (*n*=5), *Alx4* (*n*=3), *Lhx2* (*n*=2), *Prrx2* (*n*=3) and *Pkdcc* (*n*=4). For whole-mount *in situ* hybridization, *n*=3 biological replicates from three independent experiments were analyzed per gene and genotype. Scale bars: 250 µm.

The spatial distribution of 143 SMAD4 target genes downregulated at E10.0 (29-31 somites; Fig. 2D; Table S4) was analyzed by comparative WISH of wild-type and mutant forelimb buds, which yielded results for 91 genes. The genes analyzed are shown in Figs 4C and 6 (see also Figs S2 and S3). This screen revealed the reduced expression (Fig. 6A) and spatial alterations in the anterior mesenchyme of *Smad4*^Δ/Δc^ forelimb buds for target genes functioning in the BMP pathway (Fig. 6B,C). They include several regulators of BMP signaling in limb buds, such as the transcriptional sensors Id1, Id2 and Id3, Msx2 and the inhibitory SMAD proteins Smad6 and Smad7 (Fig. 6B; Zhao et al., 2000). *LacZ* reporter assays establish that for three of these BMP pathway genes (*Id1*, *Id2* and *Msx2*), the genomic regions enriched in SMAD4-chromatin complexes encode bona fide CRMs that function as transcriptional enhancers (Fig. 6C). The spatial activities of these enhancers recapitulate significant aspects of limb bud mesenchymal expression of the associated *Id1*, *Id2* and *Msx2* target genes (Fig. 6B,C). The other target genes, the spatial expression of which is reduced and altered in *Smad4*^Δ/Δc^ forelimb buds (Fig. 6A,D) function either in antero-posterior limb bud patterning (*Alx4* and *Tbx2*; Farin et al., 2013; Kuijper et al., 2005), outgrowth and/or chondrogenesis (*Sfrp2*, *Snai1*, *Lhx2* and *Prrx2*; Chen and Gridley, 2013; Geetha-Loganathan et al., 2008; Taher et al., 2011; Tzchori et al., 2009). With the exception of *Mxd4* and *Sfrp2*, these genes are part of the differentially expressed target genes shared between *Smad4*^Δ/Δc^ forelimb buds at E10.0 and E10.5 (*n*=43, Fig. S5). In particular, the early and persistent downregulation of SMAD4 target genes functioning in the BMP pathway (*Smad6*, *Smad7*, *Id1*, *Id2*, *Id3* and *Msx2*) and bone development (*Lhx2*, *Snai1*, *Pkdcc* and *Pthr1*; Imuta et al., 2009; Karaplis et al., 1994; Probst et al., 2013) indicates that the disruption of chondrogenesis and bone formation (Bénazet et al., 2012) is rooted in these early transcriptional changes (Fig. 6D, Fig. S5). The SMAD4-enriched CRMs associated with the *Alx4*, *Lhx2* and *Pkdcc* genes display robust and predominant enhancer activities in the anterior limb bud mesenchyme, while for the *Prrx2*-associated CRM, low and variable *LacZ* activity is detected in the proximal mesenchyme (Fig. 6E). With the exception of the latter, these enhancer activities and the reduced anterior expression of the associated target genes in *Smad4*^Δ/Δc^ forelimb buds (arrowheads in Fig. 6B,D) indicate that one main function of SMAD4 is the positive regulation of target genes via enhancers active in the anterior mesenchyme (Fig. 6C,E).

### SMAD4-controlled gene regulatory networks co-regulated by SHH signaling

**Fig. 7.**
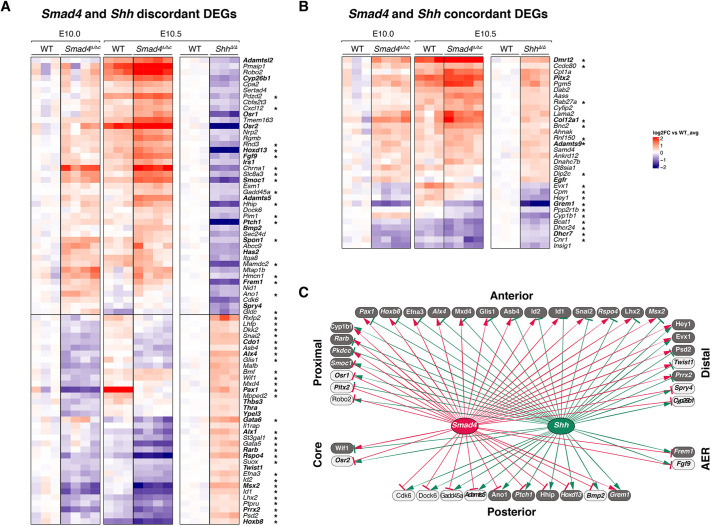
**Antagonistic SMAD4 and SHH pathway interactions control antero-posterior limb bud patterning.** (A,B) Heatmaps showing the DEGs identified by comparing wild-type (WT) and *Smad4*^Δ/Δc^ transcriptomes at E10.0 and E10.5 (wild type, *n*=3; *Smad4*^Δ/Δc^, *n*=4 biological replicates) with wild-type and *Shh*^Δ/Δ^ transcriptomes at E10.5 (wild type, *n*=3; *Shh*^Δ/Δ^, *n*=3 biological replicates). For each gene, the log2-ratio between the expression levels in each sample and the mean of the wild-type forelimb buds is shown. DEGs showing a fold-change ≥1.2 and FDR≤0.1 were analyzed (Table S14). Asterisks mark the SMAD4 target genes among the DEGs and genes indicated in bold have been linked to limb mutations. (A) *Smad4* and *Shh* discordant DEGs. (B) *Smad4* and *Shh* concordant DEGs. (C) The shared *Smad4* and *Shh* GRN consists of genes with distinct spatial expression patterns in limb buds. The shared *Smad4* and *Shh* DEGs are grouped according to their spatial ‘anterior’, ‘posterior’, ‘proximal’ or ‘distal’ expression bias (see main text). Two DEGs are expressed in the ‘core’ mesenchyme without apparent AP or PD bias and two in the AER (Table S15). The *Smad4* and *Shh* interactions within the GRN are indicated by red and green lines, respectively. Positive interactions (DEGs downregulated in *Smad4* and/or *Shh*-deficient limb buds) are represented by arrows; negative interactions (upregulated DEGs) are represented by inhibitory arrows. DEGs shown as dark-gray boxes are direct SMAD4 target genes. Genes indicated in italics have been linked to mutations causing limb skeletal phenotypes.

This molecular analysis started to uncover the SMAD4-regulated GRNs (Figs 2 and 6) that function during the transient early patterning phase that specifies digits (Zhu and Mackem, 2011; Zhu et al., 2008). During this phase, SHH signaling is required to coordinate antero-posterior and proximo-distal limb bud patterning (AP and PD patterning; Probst et al., 2011; reviewed by Zuniga, 2015). Therefore, the extent to which SMAD4-regulated genes are co-regulated by SHH signaling was determined by comparative analysis of *Smad4* and *Shh* DEGs (Probst et al., 2011) in early mouse limb buds (E10.0 to E10.5, Fig. 7A,B and Table S14). A total of 111 shared DEGs were identified and, among these, 65 are SMAD4 target genes and 37 are essential for limb development (Tissières et al., 2020). These results indicate that SMAD4 and SHH co-regulate GRNs with essential functions in early mouse limb buds. Strikingly, the majority of the shared DEGs are regulated in a discordant manner (79 genes; Fig. 7A; Table S14) and more than half are upregulated in *Smad4* deficient limb buds (upregulated, 43, downregulated, 36, Fig. 7A; Table S14). Among the SMAD4 target genes, seven are BMP pathway genes regulated in a discordant manner, which includes the BMP antagonist *Smoc1* (Fig. 7A; see also Fig. 5E and Fig. S6; Okada et al., 2011; Thomas et al., 2017). Furthermore, about half of all discordantly regulated genes are transcription factors, pointing to an important amplification of the response to BMP and SHH signaling in early limb buds. In contrast, few genes with known functions in limb bud development are present among the concordantly regulated DEGs (*n*=32, Fig. 7B, Table S14). Notable exceptions are *Grem1* and some cholesterol pathway genes (*Dhcr7*, *Dhcr24*, *Insig1* and *Cyp1b1*), which are downregulated in both *Smad4*- and *Shh*-deficient limb buds (Fig. 7B; Table S14; see also Fig. 4).

To gain insight into the interactions of these two pathways during early limb bud patterning, we screened the shared DEGs (Fig. 7A,B) for genes with distinct spatial expression patterns in mouse limb buds using the Mouse Genome Informatics and EMBRYS databases. This identified 41 DEGs with spatially restricted and asymmetrical distributions in early limb buds, which were categorized as anteriorly, posteriorly, proximally or distally expressed genes. Genes expressed more widespread were assigned to the category matching the limb bud mesenchymal region of their predominant/highest spatial expression (column ‘expression bias’ in Table S15). For example, *Msx2* is expressed anteriorly and posteriorly in wild-type mouse limb buds, but as its anterior domain is wider, *Msx2* was annotated as an anteriorly biased gene (Fig. 6B, Table S15). In contrast, *Pkdcc* was annotated as a proximal gene due to its expression by the proximal mesenchyme and exclusion from the distal mesenchyme (Fig. 6D, Table S15). Strikingly, all anteriorly biased genes are SMAD4 target genes (dark gray boxes, Fig. 7C), the expression of which is regulated positively by *Smad4* and negatively by *Shh* (Fig. 7C; *Smad4*, red activating arrows; *Shh*, green inhibitory arrows). Conversely, the majority of posteriorly biased genes are regulated negatively by *Smad4* (red inhibitory arrows) and positively by *Shh* (green activating arrows, Fig. 7C). The exception is *Grem1*, which is positively regulated by both SMAD4-mediated BMP signal transduction and SHH signaling as part of the self-regulatory feedback signaling system (Fig. 7C; Bénazet et al., 2009, 2012). In addition, *Smad4* and *Shh* have opposing effects on the majority of DEGs with asymmetrical distributions along the PD axis. However, there appears to be no predominant positive or negative regulatory impact on PD gene expression from either pathway (Fig. 7C). This analysis indicates that SMAD4-mediated BMP and SHH signaling have overall rather opposing effects on co-regulated genes that are part of the GRNs regulating AP and PD axes patterning (reviewed by Zuniga and Zeller, 2020) during early mouse limb bud outgrowth.

## DISCUSSION

The present study identifies the SMAD4 target genes in the early mouse forelimb bud mesenchyme. SMAD4-mediated BMP signal transduction is predominant during the onset of forelimb bud development (Pignatti et al., 2014). In particular, the *Bmp2*, *Bmp4* and *Bmp7* ligands are expressed at high levels and essential for forelimb bud patterning and skeletal development (Bénazet et al., 2009; Luo et al., 1995). The *Tgfb2* and *Tgfb3* ligands are also expressed at comparable levels in early forelimb buds (this study; reviewed by Lorda-Diez et al., 2021), but inactivation of *Tgfbr2* showed that TGFβ signaling is not essential for limb bud patterning (Dunker et al., 2002). In contrast, limb bud mesenchyme-specific inactivation of *Bmpr1* causes patterning defects and SMAD4-mediated BMP-signal transduction is essential for initiation of chondrogenesis (Bénazet et al., 2012; Lim et al., 2015; Ovchinnikov et al., 2006). Although crosstalk between BMP and TGFβ SMAD4-mediated signal transduction is likely (Karamboulas et al., 2010), our study indicates that SMAD4 functions mostly in BMP-signal transduction during early limb bud development as the upregulation of known targets and sensors of BMP activity, such as *Id* genes, *Msx2* and *Grem1*, is disrupted in *Smad4*-deficient limb buds (this study; Bénazet et al., 2009, 2012; Brugger et al., 2004; Hsu et al., 1998; Lopez-Rovira et al., 2002). Whereas BMP activity is high during initiation of limb bud development, it drops to lower levels during outgrowth and proliferative expansion of LMPs (Reinhardt et al., 2019). This reduction is paralleled by significant changes in the binding specificities of SMAD4-chromatin complexes and the range of DEGs and SMAD4 target genes in the forelimb bud mesenchyme (this study). However, SMAD4 target genes expressed in the anterior forelimb bud mesenchyme are likely targets of BMP4 and/or BMP7 signal transduction, as their expression overlaps these ligands.

Unexpectedly, SMAD4 is required for maintaining the expression of the majority of cholesterol biosynthesis enzymes in early limb buds, as their expression is prematurely downregulated and intracellular cholesterol reduced in *Smad4*^Δ/Δc^ forelimb buds. In addition to maternal sterols being a major source of cholesterol, defects in embryonic cholesterol synthesis cause congenital malformations similar to *Shh* loss-of-function defects (reviewed by Porter and Herman, 2011). SHH biogenesis and SMO-mediated signal transduction depend on cholesterol modification, the synthesis of which requires 20 different enzymes (reviewed by Radhakrishnan et al., 2020). Extensive follow-up analysis did not reveal any cholesterol-dependent alterations in the response to *Shh* signaling in *Smad4*^Δ/Δc^ forelimb buds and mutant LMPs. The likely reason is that the *Smad4* deficiency reduces, but does not disrupt, endogenous cholesterol biosynthesis, in contrast to mutations in the *Dhcr7* enzyme (reviewed by Horvat et al., 2011). In addition, other pathways are likely to contribute to the regulation of embryonic cholesterol synthesis. Nevertheless, our analysis shows that SMAD4 negatively regulates the expression of the SHH receptor *Ptch1* and the extracellular SHH antagonist *Hhip*, which might contribute to modulating the range of SHH signal transduction in limb buds (reviewed by Briscoe and Therond, 2013). In particular, increased *Hhip* expression might reduce SHH activity in the more central and anterior mesenchyme of *Smad4*^Δ/Δc^ forelimb buds (Chuang et al., 2003 and this study). The transcriptional regulation of cholesterol enzymes may be a more general function of SMAD4-mediated signaling because, during palate development, transcriptional profiling identified a downregulation of cholesterol synthesis enzymes and an upregulation of *Hhip* in *Tgfbr2-*deficient mouse embryos (Pelikan et al., 2013).

BMP signaling functions in multiple processes during temporal progression of limb bud development, starting with AER formation, limb bud outgrowth, chondrogenesis and ultimately in apoptosis of the interdigital mesenchyme (Pignatti et al., 2014). Based on loss- and gain-of-function studies, the BMP pathway was proposed to function downstream of SHH signaling in the limb bud mesenchyme, but at the same time inhibit SHH signaling during limb bud patterning and outgrowth (Bastida et al., 2009; Bénazet et al., 2009, 2012; Panman et al., 2006; Selever et al., 2004; Zuniga et al., 1999). Inactivation of *Bmp* ligands and their receptors in the limb bud mesenchyme causes both pre- and postaxial polydactylies, in agreement with *Bmp* expression in the anterior and posterior distal mesenchyme (Bandyopadhyay et al., 2006; Bénazet et al., 2009; Katagiri et al., 1998; Selever et al., 2004). However, the resulting limb skeletal phenotypes could be far downstream or be an indirect effect of disrupting BMP-signal transduction as a consequence of global alterations affecting morphogenetic signaling during limb bud development. Therefore, the identification of SMAD4 targets genes with morphoregulatory functions in early limb buds provides important insight into the direct impact of SMAD4 on gene expression (this study). SMAD4 regulates the expression of genes in both the anterior (including *Alx4*, *Tbx2*, *Msx2*, *Prrx2* and *Snai1*) and posterior limb bud mesenchyme (including *Hoxd13*, *Grem1*, *Bmp2*, *Cdk6*; this study and Bénazet et al., 2012; Lopez-Rios et al., 2012). Together with previous studies, our analysis reveals the direct involvement of SMAD4-mediated BMP signal transduction in the overall transcriptional upregulation of anterior and downregulation of posterior genes during early limb bud patterning (this study and Bandyopadhyay et al., 2006; Bastida et al., 2009; Bénazet et al., 2009, 2012; Ovchinnikov et al., 2006; Selever et al., 2004). Furthermore, the SMAD4 target genes *Alx4*, *Msx2*, *Prrx2* and *Dkk1* are required to restrain the developing limb bud to pentadactyly, as their genetic inactivation causes polydactyly (ten Berge et al., 1998; Kuijper et al., 2005; Lallemand et al., 2005; Mukhopadhyay et al., 2001). This indicates that SMAD4-mediated BMP signal transduction is required during the transient early phase of SHH-mediated digit patterning (Zhu et al., 2008) to maintain pentadactyly by directly regulating the expression of its target genes, many of which are co-regulated by SHH signaling. SHH is not only essential for AP digit patterning but also functions in coordinating AP and PD axes development during limb bud outgrowth (Probst et al., 2011; Zhu et al., 2008). This is of interest in light of the globally opposing transcriptional regulation by SMAD4 and SHH signaling, which reveals the direct antagonistic interactions in transcriptional regulation of genes with AP and PD expression bias and functions. SHH signaling upregulates the expression of posterior genes and prevents posterior expansion of anterior SMAD4 target genes. In the anterior mesenchyme, BMP signals via SMAD4 to upregulate and/or maintain the expression of SMAD4 target genes independently of SHH signaling, while preventing anterior expansion of posterior genes, with the exception of the BMP antagonist *Grem1*. *Grem1* is a transcriptional node in the self-regulatory signaling system that integrates transacting inputs from both SMAD4-mediated BMP and GLI-mediated SHH signal transduction into the dynamic spatio-temporal regulation of its transcription expression (Malkmus et al., 2021). The increase in GREM1-mediated BMP antagonism is balanced by feedback regulation, which results in persistent low levels of BMP activity within the posterior limb bud mesenchyme (Bénazet et al., 2009; Malkmus et al., 2021). Low-level BMP activity is relevant to restrain the autopod to pentadactyly as transgene-mediated *Grem1* overexpression results in polydactyly (ten Berge et al., 1998; Kuijper et al., 2005; Lallemand et al., 2005; Mukhopadhyay et al., 2001; Norrie et al., 2014). Most importantly, GREM1-mediated BMP antagonism in the posterior mesenchyme is required to establish and propagate the self-regulatory feedback signaling system that enables distal progression of limb bud outgrowth and patterning (Zuniga, 2015; Zuniga et al., 1999). The intricacy of the direct molecular interactions is further exemplified by the fact SMAD4 directly controls the transcription of two out of the three BMP ligands, *Bmp2* and *Bmp4*, and the BMP antagonists *Grem1* and *Smoc1*, which are essential for normal limb bud development. The analysis of SMAD4-regulated genes (DEGs) and target genes leads us to propose that BMP activity is maintained at high levels in the anterior mesenchyme, which is supported by the lack of BMP antagonist expression in this territory (*Smoc1* and *Grem1*, this study and Okada et al., 2011; Zuniga et al., 1999). During pectoral fin bud development, BMP signaling gradients are important for fin bud growth, and these gradients are modulated by SMOC1, which reinforces the importance of tight modulation of BMP activities during morphogenesis (Mateus et al., 2020).

Mouse genetic experiments and experimental manipulation of chicken limb buds have shown that tampering with BMP activity levels alters *Shh* expression (Bastida et al., 2009; Bénazet et al., 2009, 2012; Norrie et al., 2014). Our analysis shows that this is likely indirect, as *Shh* expression and signal transduction are not affected in *Smad4*-deficient mouse limb buds. Rather unexpectedly, our study identifies the Hedgehog inhibitory gene *Hhip* as a negatively regulated SMAD4 target gene, which indicates that SMAD4-mediated signal transduction not only modulates BMP activity but also participates in fine-tuning the SHH activity range in limb buds. Thus, SMAD4-mediated BMP signal transduction and SHH signaling have both direct opposing impacts (posterior mesenchyme) and coordinated interactions (anterior limb bud mesenchyme; Bastida et al., 2009; Bénazet et al., 2009; Dunker et al., 2002). This highlights the intricate control of morphogenetic signaling by BMPs and SHH during the early phase that is crucial to digit specification and patterning in mouse limb buds (Zhu et al., 2008). In the neural tube, high BMP activity is required for dorsal neural tube patterning, while, ventrally, SHH and the BMP antagonist chordin are co-expressed by the notochord, and the interaction of both secreted factors is required to induce the ventral floor plate (Patten and Placzek, 2002). In fact, *Shh* and *Bmp* ligands are co-expressed in numerous tissues, indicating that the interactions of the two pathways could be conserved during embryogenesis (Bitgood and McMahon, 1995). Functionally relevant BMP and SHH signaling interactions have been reported for other developing tissues, such as tooth and mandibular arch development (Harris et al., 2002; Li et al., 2015; Madison et al., 2005; Xu et al., 2019). In contrast to these previous studies, we establish that a large fraction of the genes in the shared SMAD4-SHH GRN are direct transcriptional targets of SMAD4-mediated BMP signal transduction during early limb bud patterning and outgrowth.

## MATERIALS AND METHODS

### Ethics statement, mouse strains and embryos

All experiments conducted with Swiss Albino mice (*Mus musculus*) and embryos of both sexes at the developmental ages indicated were performed strictly respecting Swiss laws, the 3R principles and the principles of the Basel Declaration. All animal studies were evaluated and approved by the Regional Commission on Animal Experimentation and the Cantonal Veterinary Office of the city of Basel (license 1950). To conditionally inactivate *Smad4* in the forelimb bud mesenchyme, the *Prrx1-Cre* strain was used (Logan et al., 2002; Yang et al., 2002). *Prrx1-Cre^Tg/Tg^; Smad4*^Δ*/+*^ males were crossed with *Smad4^flox/+^* females to obtain experimental embryos that carry a constitutive *Smad4* null allele and a conditionally inactivated *Smad4* allele (*Prrx1-Cre^Tg/+^; Smad4*^Δ*/*Δ*c*^, referred to as *Smad4*^Δ*/*Δ*c*^). Resulting *Prrx1-Cre^Tg/+^; Smad4^+/+^* littermates were used as controls for all experiments (referred to as ‘wild type’ in the text). *Shh*^Δ/Δ^ embryos were obtained from crossing mice heterozygous for the *ShhCre* allele (Harfe et al., 2004).

### Generation of the *Smad4*^3xF^ mouse allele

The *Smad4*^3xF^ mouse strain was generated by introducing a 3xFLAG epitope tag in the endogenous SMAD4 protein by conventional homologous recombination in mouse ES cells. The used targeting vector consisted of two homology arms flanking the 3′-end of *Smad4* coding sequence, in which the 3xFLAG was inserted in frame between the exon 12 and the 3′UTR. A floxed *neomycin* selection cassette was inserted downstream of the *Smad4* locus in the 3′ homology arm. This targeting vector was linearized and electroporated in R1 mycoplasm-free embryonic stem (ES) cells (obtained from Dr Nagy, Samuel Lunenfeld Research Institute, Toronto, Canada; Nagy et al., 1993) to generate the *Smad4*^3xF^-Neo allele. ES cell clones were screened for correct recombination by Southern blot analysis using probes located either outside of the 5′ or 3′ homology arms (5′ and 3′probes) and for the *neomycin* cassette to exclude ES cell clones with random integration of the targeting construct. Correctly targeted ES cells clones were then injected in C57BL/6 blastocysts by the Centre of Transgenic Mice (CTM) of the University of Basel. Chimeric males were obtained from three independent ES cell clones and mated with *CMV-*Cre females (C57BL/6 background) to delete the floxed *neomycin* selection cassette. Germline transmission was assessed using PCR genotyping. *CMV*-Cre-mediated deletion of the floxed *neomycin* selection cassette generated the *Smad4*^*3xF*^ allele. Specific primers were used to discriminate between the *Smad4^+/+^, Smad4*^*3xF-Neo*^ and *Smad4^3xF^* alleles: PCR_Smad4^3xF^_Forward, 5′(P1)-ACAGCCTCCACACTTGTGCT-3′; PCR_Smad4^3xF^_Reverse, 5′(P2)-TGTCTGCTAAGAGCAAGGCA-3′; PCR_Smad4^3xF-Neo^_Forward, 5′(P3)-AGGACTTTCCCATGGACACTG-3′; and PCR_Smad4^3xF-Neo^_Reverse, 5′(P4)-AGCACTGCCTGGTCAGATGA-3′.

### SMAD4-3xFlag ChIP-seq

Two independent biological replicates were used to ensure reproducibility (see ENCODE guidelines https://www.encodeproject.org/about/experiment-guidelines/). About 80 *Smad4^3xF/3xF^* embryos at E9.5-E10.0 (25 to 30 somites; forelimbs with proximal trunk tissues) or 100 *Smad4^3xF/3xF^* embryos E10.5 (forelimbs/hindlimbs) were dissected per replicate. The ChIP protocol was performed as previously described (Osterwalder et al., 2014) with one modification: SMAD4-chromatin complexes were immunoprecipitated for only 6 h instead of overnight to reduce non-specific background. Libraries for sequencing were constructed using the KAPA Hyper Prep Kit (ref KK8502) and sequenced using the Illumina NextSeq 500 system.

### ChIP-seq analysis and annotation

Short reads obtained from Illumina NextSeq were aligned to the mm9 genome using Bowtie v1.1.0 (Langmead et al., 2009). Only those reads with a unique match to the genome with two or fewer mismatches (*-m 1 -v 2*) were retained. In order to make different runs comparable, the 3′ of reads were trimmed to 63 bp before alignment. This step was performed using fastx_trimmer (*-l 63*), a tool part of the FASTX-Toolkit (http://hannonlab.cshl.edu/fastx_toolkit/) (v0.0.13). Peak calling was performed using MACS v1.4 (Zhang et al., 2008) with the following parameters: *--gsize=mm --bw=300 --nomodel --shiftsize=100 --pvalue=1e-2*. Input DNA from the same sample was used as a control. Wiggle tracks were also generated with MACS; these were then re-scaled linearly according to sequencing depth (RPM, Reads Per Million sequenced reads). MACS was run with a permissive threshold (*P*-value 0.01) in order to identify a larger list of sub-significant regions across biological replicates. Evidences from these replicates were combined using MSPC (Jalili et al., 2015), with the following parameters: *-r biological -s 1E-5 -W 1E-2*. The confirmed peaks were assigned the best *P*-value (as defined by MACS) among the overlapping peaks across replicates. Only replicated peaks were retained for further analysis (termed as *golden* for convenience; one golden set per developmental stage). These lists of peaks were annotated to the TSS of the nearest RefSeq genes using the script *annotatePeaks.pl* available in HOMER (Heinz et al., 2010). A region was considered as proximal to a promoter if located within 2.5 kb of a RefSeq promoter. The remaining regions were divided into intragenic and intergenic, whether the region overlapped the body of an annotated gene or not.

### Motif enrichment and *de novo* motif discovery analyses

The script *findMotifsGenome.pl* available in HOMER (Heinz et al., 2010) was used to perform enrichment analysis for known transcription-factor binding sites and motif discovered *de novo*. The script was run with the following arguments: *-size -150,150 -len 6,7,8,9,10,12,14*, using the peak summits of the *golden* set as reference. The top ten most significant, over-represented known matrices along with the top ten *de novo* discovered motifs were then used to scan every single region for high-affinity sites using FIMO (v4.10.0; Grant et al., 2011). The following parameters were used: *--thresh 1e-4 --no-qvalue*. The resulting list of sites was transformed into a matrix in which each region was represented as a vector of *P*-values, one for each different motif, corresponding to the *P*-value of the highest-scoring site identified (*P*-value=1 if no significant match was found). *P*-values were then log10-transformed and their sign inverted, then hierarchically clustered (*hclust* function of R; Euclidean distance; complete linkage).

### Evolutionary conservation analysis of the genomic regions enriched in SMAD4-chromatin complexes

The genome-wide track of base-pair *Phastcons* (Siepel et al., 2005) conservation scores in placental mammals was downloaded from the UCSC genome browser (Tyner et al., 2017) (track name: *mm10.60way.phastCons60wayPlacental.bw*). The coordinates of the peaks in the golden sets were converted from mm9 to mm10 using *liftOver* (Tyner et al., 2017) (*-minMatch=0.95*). The base-pair scores for the 300 bp centered on the summit of the peaks were then extracted using *bwtool* (Pohl and Beato, 2014).

### ChIP-qPCR analysis

Two BMP responsive elements (BRE of *Msx2* and BRE of *Id1*; Brugger et al., 2004; Korchynskyi and ten Dijke, 2002) identified in the ChIP-seq dataset were validated by ChIP-qPCR. Each duplicate contains 45 pairs of forelimbs with proximal trunk tissues from *Smad4^3xF/3xF^* embryos at E9.75 or fore- and hindlimbs buds at E10.5. An unlinked amplicon within the *β-actin l*ocus was used as a normalizing control and to calculate the fold-enrichment. A qPCR cycle threshold of 32 was defined as background enrichment. For each experiment, two genomic regions not enriched in the SMAD4^3xF^ ChIP-seq dataset were used as negative controls. These are oligos used for qPCR amplification: ChIPqPCR_BRE Id1-Forward, 5′-AGAATGCTCCAGCCCAGTTT3′; ChIPqPCR_BRE Id1-Reverse, 5′-TGACGTCACCCATTCATAAAA-3′; ChIPqPCR_BRE Msx2 Forward, 5′-CCATTAGGGCGAATTGTCAT-3′; ChIPqPCR_BRE Msx2-Reverse, 5′-GAGCCGCGTTAATTGCTCT-3′; ChIPqPCR_Neg Id1-Forward, 5′-TTCTTCTCTGGCTGCCAGTG-3′; ChIPqPCR_Neg Id1-Reverse, 5′-AACTGAGCCTTGCATCATGC-3′; ChIPqPCR_Neg Msx2-Forward, 5′-GACTAGGGCTCTCTTTTCCTGA-3′; ChIPqPCR_Neg Msx2-Reverse, 5′-CATTTCTCCACCCCAGCTTA-3′; ChIPqPCR_β-actin-Forward, 5′-GATCTGAGACATGCAAGGAGTG-3′; and ChIPqPCR_β-actin-Reverse, 5′-GGCCTTGGAGTGTGTATTGAG-3′.

### ATAC-seq analysis and annotation

Two independent biological replicates (*n*=2) were generated to determine reproducible signals, see, for example https://informatics.fas.harvard.edu/atac-seq-guidelines.html. For the early stage, each replicate contains a pair of forelimbs with proximal trunk tissues isolated from wild-type embryos at E9.75. For the later stage, each replicate consists of a pair of forelimbs isolated from wild-type embryo at E10.5. For both stages, two biological replicates were processed independently as described previously (Buenrostro et al., 2013). The ATAC libraries were prepared by amplifying the transposed DNA fragments with the KAPA HiFI HotStart ReadyMix kit followed by sequencing on an Illumina NextSeq 500. The short reads were aligned to the mm9 genome using Bowtie v1.1.0 (Langmead et al., 2009; -m 1 -v 2, see section ‘ChIP-seq analysis and annotation’). Accessible regions were identified using MACS v1.4 (Zhang et al., 2008) with the following parameters: *--gsize=mm --bw=150 --nomodel --nolambda --shiftsize=75*. Genome-wide profiles were generated using MACS and re-scaled linearly according to sequencing depth (RPM). Gene annotation was performed using HOMER (Heinz et al., 2010), as described in the section ‘ChIP-seq analysis and annotation’. Evidence from biological replicates was combined using MSPC (Jalili et al., 2015), using the following parameters: *-r biological -s 1E-10 -W 1E-6*. The confirmed regions were assigned the best *P*-value (as defined by MACS) among the overlapping regions across replicates.

### RNA-seq analysis

Dissected wild-type and *Smad4^Δ/Δc^* forelimb buds from E10.0 embryos (30 somites) and 10.5 embryos (35 somites) were collected in RNAlater (Sigma R0901), incubated overnight at 4°C and then stored at −80°C. Both forelimb buds of one biological replicate were pooled. In principle, the accepted sample size in the field is *n*=2 biological replicates to ensure reproducibility; see ENCODE guidelines https://www.encodeproject.org/about/experiment-guidelines/. After genotyping, four age-matched and gender-matched *Smad4^Δ/Δc^* forelimb bud pairs and three wild-type replicates per stage were sequenced. RNA was extracted using the Qiagen RNeasy micro kit. For each replicate, the quality of total RNA was analyzed using the RNA 6000 Pico kit (Agilent 2100 bioanalyzer), which was followed by polyA-mediated RNA library preparation. Sequencing was carried out on a HiSeq 2500 machine using the single-read 50 cycles protocol.

Single-end reads obtained from Illumina HiSeq were aligned to the mm9 reference genome and to the *Mus musculus* transcriptome (iGenome refGene GTF) using TopHat v2.0.13 (Kim et al., 2013). The option *--no-coverage-search* was specified, whereas all the other parameters were left to default. Only uniquely mapped reads were considered for the analysis. Tracks for the UCSC genome browser (Tyner et al., 2017) were produced using *genomeCoverageBed* from BedTools v2.17.0 (Quinlan and Hall, 2010); these were linearly re-scaled according to sequencing depth (RPM). Gene-wise counts were computed using *htseq-count* from the HTSeq package (Anders et al., 2015) with *-s* set to *no*. Genes on chromosomes X, Y and M were excluded from further analysis. edgeR (Robinson et al., 2010) was used to identify DEGs. Only genes showing expression (in terms of fragments per million sequenced reads equal or higher than 1) in at least three samples were considered for further analyses. Libraries were normalized according to TMM normalization. Tag-wise estimation of dispersion was evaluated using *prior.*d.f.*=10*. Differential expression between pairs of conditions was evaluated using the *exactTest* R function. False discovery rates were estimated using Benjamini-Hochberg correction (Benjamini and Hochberg, 1995). DEGs were defined as those genes showing a *q*-value≤0.1 and a linear fold-change equal or higher than 1.2. Functional enrichment analyses were conducted using DAVID (Huang et al., 2009).

The SMAD4-bound regions were associated with their target genes using the TADs defined in mouse ES cells (Dixon et al., 2012). At a particular developmental stage, the expressed genes were classified as either unchanged, upregulated or downregulated. Each gene was assigned to the corresponding TAD, and the number of SMAD4-binding peaks per TAD was calculated and normalized to the total number of genes within the domain. Using this strategy, it was possible to assign the SMAD4-interacting genomic regions to particular genes.

### Hierarchical clustering, plots and statistical testing

Clustering, plots, heat maps and statistics were handled in the statistical computing environment R v3. The GO Enrichment Analysis plots in Figs 2D and 3D were generated with the Top 20 enriched GO Biological Process Terms (among those with FDR≤0.05) as inferred by http://geneontology.org/ release 2021-09-01 (Ashburner et al., 2000; Gene Ontology Consortium, 2021; Mi et al., 2019).

### Immunofluorescence analysis

Embryos were collected in ice-cold PBS and fixed for 2 h at 4°C in 4% PFA/PBS. Samples were then cryoprotected using a gradient of sucrose: 10% sucrose/PBS (w/v), 20% sucrose/PBS and 30% sucrose/PBS (1 h each) at 4°C. Embryos were then embedded 50:50 (v/v) OCT/30% sucrose. For immunofluorescent staining, 10 µm sections were prepared. *Smad4^3xF/3xF^* or wild-type sections were washed for 3×5 min in PBS, once 30 min in PBT and again 5 min in PBS. They were blocked in 1% BSA in PBT for 1 h at room temperature and incubated overnight at 4°C with the monoclonal mouse anti-FLAG M2 antibody (Sigma, F1804) diluted 1:500 in 1% BSA/PBS (Osterwalder et al., 2014). Sections were washed for 3×5 min in PBS, once in PBT and were incubated in the dark for 1 h at room temperature with the goat anti-mouse Alexa 488 secondary antibody (Invitrogen, A-11001) diluted 1:500 in 1% BSA/PBS (Osterwalder et al., 2014). Sections were finally washed for 3×10 min in PBS, once in PBT (5 min), nuclei were counterstained in 1 µg/ml Hoechst-33258/PBS (5 min) and sections were rinsed again for 3×5 min in PBS. They were then mounted in Mowiol 4-88 and dried overnight at room temperature in the dark.

### Whole mount *in situ* hybridization (WISH)

*n*≥3 independent biological samples were analyzed. Gene expression patterns in embryos are extremely robust and, based on our previous experience (Bénazet et al., 2009) and the standard in the field, no variability is observed between embryos of the same stage. Embryos were age-matched by counting somites. Whole-mount *in situ* hybridization was performed using standard protocols. *Smad4^+/+^; Prrx1-Cre^Tg/+^* embryos were always used as wild-type controls.

### Generation and analysis of transgenic *LacZ* founder embryos

The aimed sample size that is standard in the field to determine tissue-specific enhancer activity is *n*≥3 independent transgenic embryos expressing the LacZ reporter in the tissue of interest (Visel et al., 2007). CRM regions were amplified by PCR from mouse genomic DNA and were then cloned into a Hsp68-*LacZ* reporter vector (Osterwalder et al., 2014) using the Gibson Assembly Method. Transgenic embryos were generated by pronuclear injection.

### Culture of wild-type and mutant LMPs in cholesterol-free medium and quantitation of intracellular cholesterol

Forelimb buds (E10.0, 28-29 somites) were collected into ice-cold PBS and incubated in cold 2% Trypsin (Gibco 15090-046)/PBS at 4°C for 30 min. The reaction was stopped by adding an excess of DMEM medium containing 10% fetal bovine serum (FBS). The limb bud ectoderm was then removed. By gently pipetting, LMPs were dissociated and seeded in two or three wells of a 96-well plate with DMEM medium containing 10% lipid-depleted FBS (PanBiotech P30.3302), 4.5 g/l glucose (Gibco 41966-029), 100 U penicillin, 0.1 mg/ml streptomycin (Sigma P-0781) and 200 mM L-glutamine (Sigma G-7513). LMPs were cultured in this cholesterol-free medium for 20 to 24 h and then treated with 10 mM methyl-β-cyclodextrin (MβCD, Sigma C4555) for 15 min at 37°C to remove cholesterol from plasma membranes. Then, LMPs were trypsinized gently for 2 min in 2% trypsin and LMPs from two pairs of forelimb buds of the same genotype pooled for one biological replicate. After determining cell numbers, LMPs were mixed with 2 ml of ethanol/chloroform solution (2:1) in a glass tube. Following 5 min centrifugation at 1400 ***g***, the supernatant was transferred into a new glass tube and mixed with 250 µl 50 mM citric acid, 500 µl water and 250 µl chloroform. After 30 min centrifugation at 1400 ***g***, the lowest phase-containing lipids was transferred in a 1.5 ml Eppendorf tube and dried using a Speedvac centrifuge. The dried lipids were solubilized in 95% ethanol and the cholesterol was quantitated using the Amplex Red Cholesterol Assay kit (Invitrogen A12216). All values for the wild-type and mutant samples are shown in Table S9. All measured values for the wild-type samples were clearly above the detection limit. The concentrations of intracellular cholesterol were calculated by dividing the measured cholesterol levels by the cell numbers determined.

Preparation of lipid/cholesterol depleted fetal bovine serum (FBS): 500 ml FBS were stirred overnight at 4°C with 10 g Cab-osil M-5 (ACROS Organics 7631-86-9). The mix was then centrifuged for 10 min at 2500 ***g*** and the supernatant filtrated under sterile conditions. The Insulin-Transferrin-Sodium-Selenite media supplement (Sigma I-1884-1) was dissolved in 50 ml of H_2_O, acidified by adding 250 µl HCl and filtrated under sterile conditions. 25 ml of the Insulin-Transferrin-Sodium-Selenite solution was added to 500 ml of lipid-depleted FCS. Aliquots of 30 ml were frozen at −20°C and used for preparing 500 ml of lipid-depleted EMFI medium.

### Imaging

Images were taken using a Leica MZ FLII stereomicroscope and the Leica Application Suite V3 software. Contrast and image size were adjusted with Adobe Photoshop CS5.1. All bar plots were generated using GraphPad Prism 7. All figures were generated with Adobe Photoshop or Adobe Illustrator.

### Statistical analysis

#### ChIP-seq

After sequence alignment, peak calling was performed using MACS v1.4 (Zhang et al., 2008) with a *P*-value threshold of 1e-2.

#### RNA-seq

Following the alignment of sequences, edgeR (Robinson et al., 2010) was used to identify DEGs. DEGs were defined as those genes showing an FDR≤0.1 and an absolute linear fold-change equal or higher than 1.2.

### Databases used for analysis

The gene expression annotations for the network shown in Fig. 7C were made using the gene expression pattern databases of the Mouse Genome Informatics (http://www.informatics.jax.org) and EMBRYS (https://www.embrys.jp).

## Supplementary Material

Supplementary information

Reviewer comments
