# Supplementary material for: SMAD4 target genes are part of a transcriptional network that integrates the response to BMP and SHH signaling during early limb bud patterning
Source: Development. 2021 Dec 3;148(23):dev200182. doi: 10.1242/dev.200182 (PMC8714076; doi:10.1242/dev.200182)
Supplement: Supplementary information [file develop-148-200182-s1.pdf]

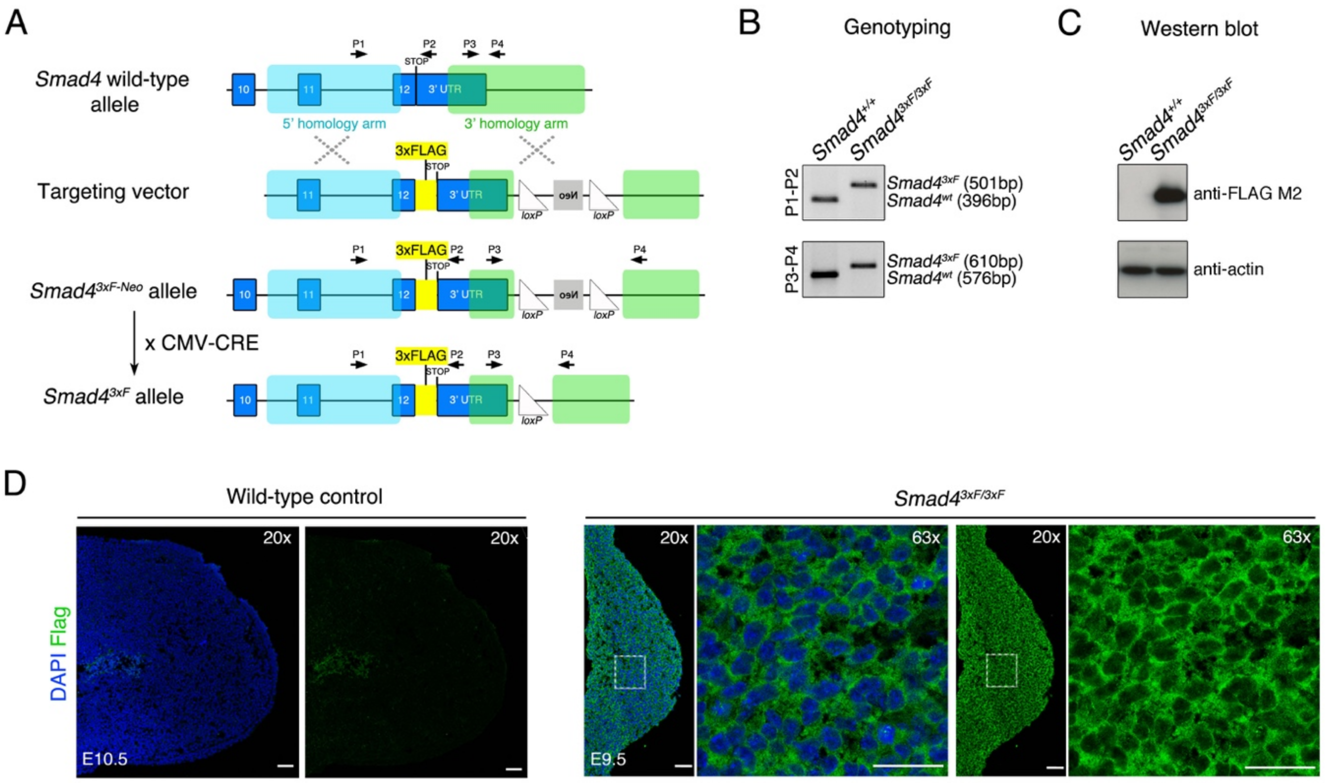

Fig. S1. Generation of the *Smad4*^{3xFlag} *allele*

(A) The *Smad4*^{3xF} allele was generated by homologous recombination in mouse ES cells. A vector containing two homology arms flanking the 3' end of the *Smad4* coding sequence and a 3xFLAG epitope tag was inserted in frame between coding exon 12 and the 3'UTR of the *Smad4*^{wt} allele. The targeting vector also contains a floxed Neo cassette downstream of the *Smad4* sequence. Initially, *Smad4*$^{3xF\text{-}Neo}$ mice were crossed with *CMV*-CRE mice to remove the Neo selection cassette, which generated the *Smad4*^{3xF} allele (ΔNeo). The arrows indicate the primers used for genotyping. (B) Genotyping and validation of the different *Smad4* alleles by PCR. The sizes of the diagnostic PCR bands are indicated on the right. (C) Detection of the SMAD4^{3xF} protein by Western blot in *Smad4*$^{3xF/3xF}$ forelimb buds at E11.5. (D) Fluorescent immunostaining using the mouse anti-Flag antibody on frozen sections of WT and *Smad4*$^{3xF/3xF}$ forelimb buds at the stages indicated (n=3). Scale bar: 100μm (High magnification: 50μm).

Development • Supplementary information

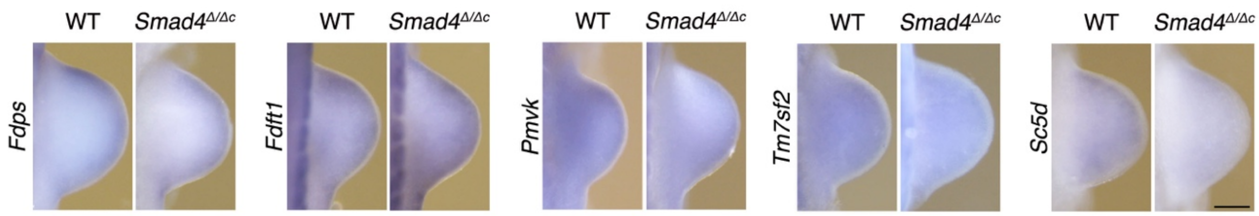

Fig. S2. The spatial expression of cholesterol biosynthesis enzymes. Comparative analysis of the spatial distribution of the transcripts for four additional genes functioning in cholesterol biosynthesis in wild-type and *Smad4*$^{\Delta/\Delta c}$ forelimb buds at E10.0 (28-31 somites). Their transcript levels are reduced in *Smad4*$^{\Delta/\Delta c}$ forelimb buds in comparison to wild-type limb buds. *Fdft1*, *Pmvk, Tm7sf2* and *Sc5d* are expressed rather uniformly, which renders detection of spatial differences more difficult. For each gene, minimally 3 independent embryos per genotype were analysed. Scale bars: 250μm.

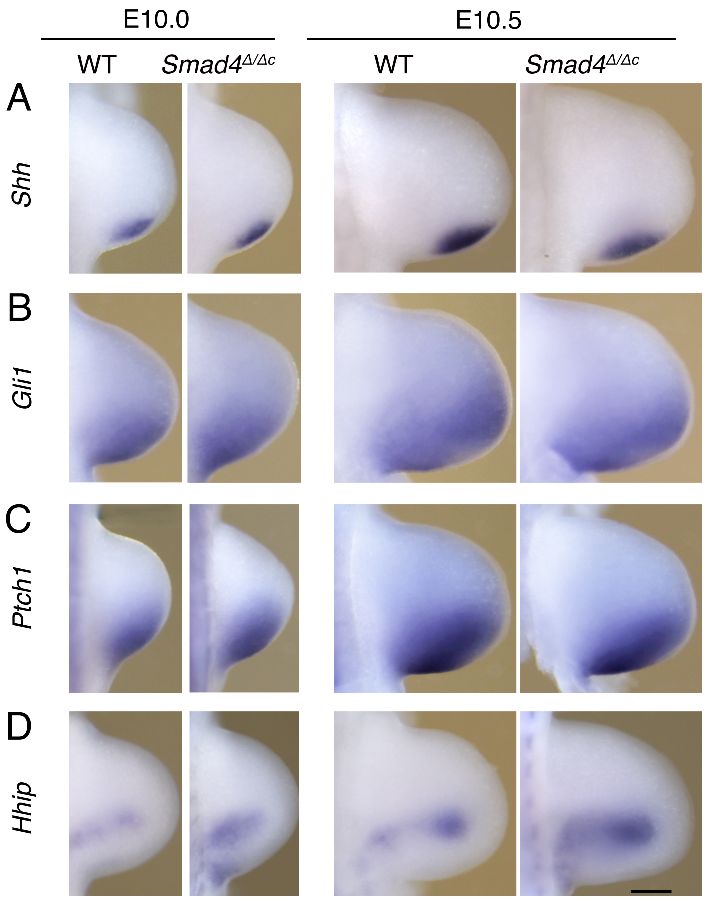

Fig. S3. The spatial expression of*Shh* and its transcriptional targets*Gli1* and *Ptch1* are not altered in *Smad4*$^{\Delta/\Delta c}$ forelimb buds.

Comparative spatial expression analysis of wild-type (WT) and *Smad4*$^{\Delta/\Delta c}$ forelimb buds at E10.0 (29-30 somites) and E10.5 (34-35 somites), n=3 independent samples were analysed per stage and genotype. (A) Spatio-temporal distribution of *Shh* in forelimb buds. (B, C) Spatial distribution of the SHH targets *Gli1* and *Ptch1*, which serve as transcriptional sensors of SHH signal transduction in responding limb bud mesenchymal cells. No significant alterations in their spatial distributions are detected, which indicates that the cellular response to SHH signal transduction is not altered. Note that *Ptch1* is also a putative SMAD4 target gene, whose transcript levels are slightly increased in *Smad4*$^{\Delta/\Delta c}$ forelimb buds at E10.0 (Table S 3). (D) Up-regulation of the SMAD4 target gene *Hhip* in the posterior mesenchyme in proximity to the *Shh* expression domain in *Smad4*$^{\Delta/\Delta c}$ forelimb buds at E10.5. The differences were seen reproducibly (n≥3 independent samples). Scale bar: 250μm.

Development • Supplementary information

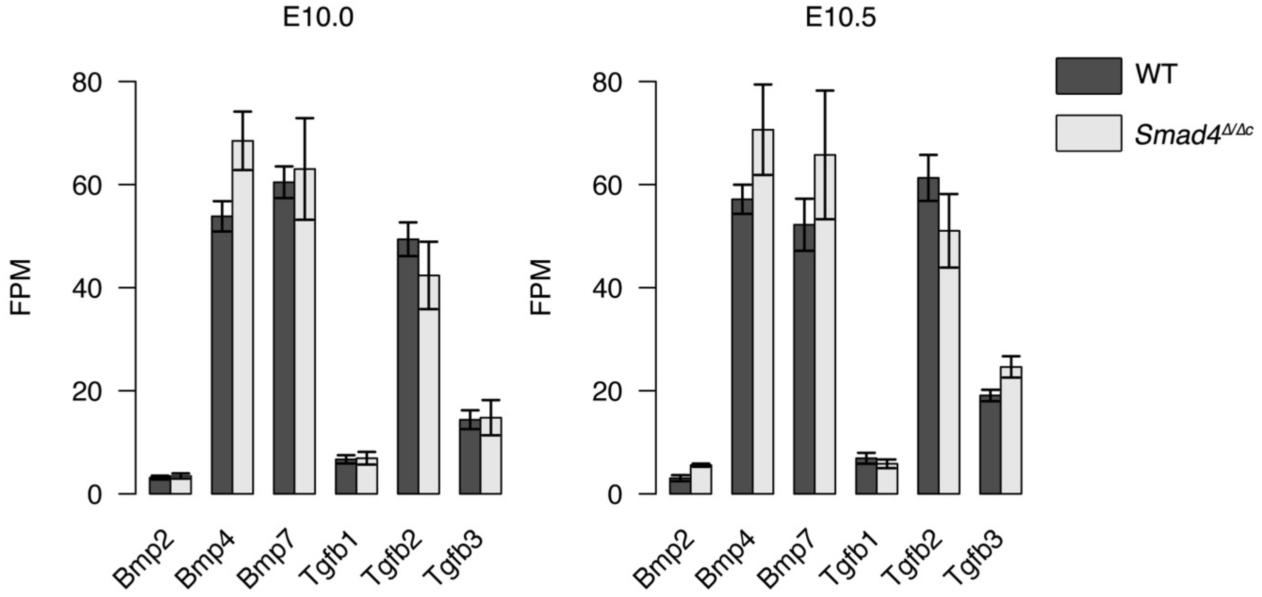

Fig. S4. Expression of the key *Bmp* and *Tgfβ* ligands in wild-type and *Smad4*$^{\Delta/\Delta c}$ forelimbs at E10.0 and E10.5.

Bar plots of the gene specific fragments per million mapped fragments (FPM; from the primary RNA-seq datasets) representing the expression of the key BMP ligands (*Bmp2, 4 and 7*) and TGFβ ligands (*Tgfb1, 2 and 3*) in forelimb buds at E10.0 (30 somites, left panel) and E10.5 (35 somites, right panel). Dark grey: wild-type (WT) FPM counts; light grey: *Smad4*$^{\Delta/\Delta c}$ FPM counts for the specific gene (WT: n=3, *Smad4*$^{\Delta/\Delta c}$: n=4 independent samples analysed).

Development • Supplementary information

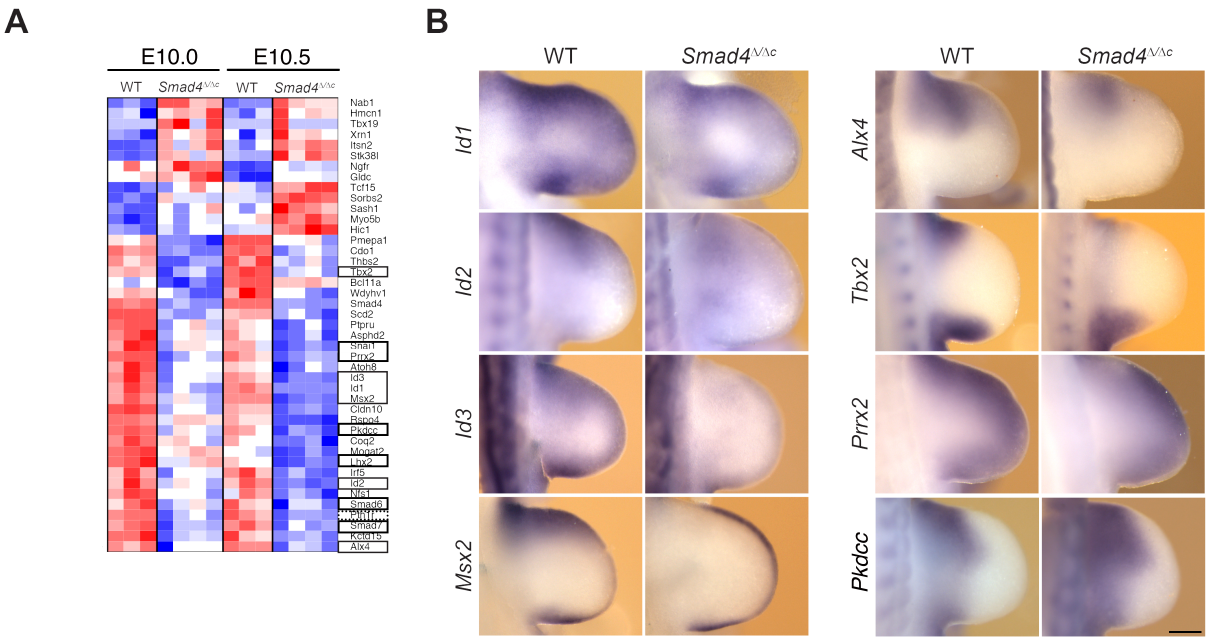

Fig. S5. The spatial expression of select shared SMAD4 target genes in forelimb buds at E10.5.

(A) Heatmap of the 42 SMAD4 target genes in forelimb buds that shared between stages E10.0 and E10.5. For each gene, the log2-ratio of the transcript levels in each replicate and the mean (white) of the three biological replicates for wild-type (WT) forelimb buds is shown. Red: increased expression; blue: reduced expression. Black boxes highlight the genes that show spatial expression changes in *Smad4*$^{\Delta/\Delta c}$ forelimb buds at either of the two stages (see also Fig. 6). The box with broken lines indicates the *Pthr1* gene (see main text). (B) Comparative expression analysis of select SMAD4 target genes in WT and *Smad4*$^{\Delta/\Delta c}$ forelimb buds at E10.5 (34-36 somites, n=3 independent samples were analysed per gene and genotype). Scale bar: 250μm.

Development • Supplementary information

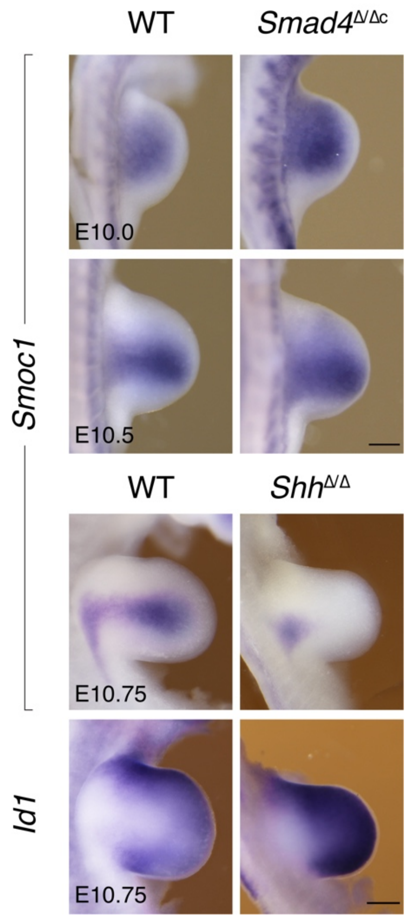

Fig. S6. *Smoc1* and *Id1* are SMAD4 target genes that are discordant *Smad4* and *Shh* DEGs. Expression analysis of SMAD4 target genes *Smoc1* in WT, *Smad4*$^{\Delta/\Delta c}$ and *Shh*$^{\Delta/\Delta}$ and *Id1* in *Shh*$^{\Delta/\Delta}$ forelimb buds between E10.0 and E10.75 (29 to 37 somites). n=3 independent samples were analysed per gene and genotype. Scale bar: 250μm.

Table S1. Genes up-regulated at least 1.2-fold in Smad4Δ/Δc compared to wild-type limb buds at E10.0

Click here to download Table S1

Table S2. Genes down-regulated at least 1.2-fold in Smad4Δ/Δc compared to wild-type limb buds at E10.0

Click here to download Table S2

Development • Supplementary information

Table S3. SMAD4 targets up-regulated at least 1.2-fold in Smad4Δ/Δc compared to wild-type limb buds at E10.0

Click here to download Table S3

Table S4. SMAD4 targets down-regulated at least 1.2-fold in Smad4Δ/Δc compared to wild-type limb buds at E10.

Click here to download Table S4

Table S5. Genes up-regulated at least 1.2-fold in Smad4Δ/Δc compared to wild-type limb buds at E10.5

Click here to download Table S5

Table S6. Genes down-regulated at least 1.2-fold in Smad4Δ/Δc compared to wild-type limb buds at E10.5

Click here to download Table S6

Table S7. SMAD4 targets up-regulated at least 1.2-fold in Smad4Δ/Δc compared to wild-type limb buds at E10.

Click here to download Table S7

Table S8. SMAD4 targets down-regulated at least 1.2-fold in Smad4Δ/Δc compared to wild-type limb buds at E10.

Click here to download Table S8

Table S9. Intracellular cholesterol levels in wild-type and Smad4Δ/Δc mutant LMPs at E10.0

Click here to download Table S9

Table S10. Genes up-regulated at least 1.2-fold in Smad4Δ/Δc compared to wild-type limb buds at both E10.0 and E10.5

Click here to download Table S10

Table S11. Genes down-regulated at least 1.2-fold in Smad4Δ/Δc compared to wild-type limb buds at both E10.0 and E10.5

Click here to download Table S11

Table S12. SMAD4 targets up-regulated at least 1.2-fold in Smad4Δ/Δc compared to wild-type limb buds at both E10.0 and E10.5

Click here to download Table S12

Table S13. SMAD4 targets down-regulated at least 1.2-fold in Smad4Δ/Δc compared to wild-type limb buds at both E10.0 and E10.5

Click here to download Table S13

Table S14. Genes differentially expressed at least 1.2-fold compared to wild type in both Smad4Δ/Δc and ShhΔ/Δ mutant limb buds at E10.0-E10.5

Click here to download Table S14

Table S15. Genes differentially expressed at least 1.2-fold compared to wild type in both Smad4Δ/Δc and ShhΔ/Δ mutant limb buds and that have regionalised expression patterns in wild type limb buds at E10.0-E10.5

Click here to download Table S15

Development • Supplementary information
