# Supplementary material for: SMAD4 target genes are part of a transcriptional network that integrates the response to BMP and SHH signaling during early limb bud patterning
Source: Development. 2021 Dec 3;148(23):dev200182. doi: 10.1242/dev.200182 (PMC8714076; doi:10.1242/dev.200182)
Supplement: Reviewer comments [file develop-148-200182_review_history.pdf]

SMAD4 target genes are part of a transcriptional network that integrates the response to BMP and SHH signaling during early limb bud patterning

Julie Gamart, Iros Barozzi, Frédéric Laurent, Robert Reinhardt, Laurène Ramos Martins, Thomas Oberholzer, Axel Visel, Rolf Zeller and Aimée Zuniga
DOI: 10.1242/dev.200182

Editor: Liz Robertson

Review timeline

Original submission:	8 September 2021
Editorial decision:	5 October 2021
First revision received:	25 October 2021
Accepted:	3 November 2021

Original submission

First decision letter

MS ID#: DEVELOP/2021/200182

MS TITLE: SMAD4 target genes are part of a transcriptional network that integrates the response to BMP and SHH signaling during early limb bud patterning

AUTHORS: Julie Gamart, Iros Barozzi, Frédéric Laurent, Robert Reinhardt, Laurène Ramos Martins, Thomas Oberholzer, Axel Visel, Rolf Zeller, and Aimée Zuniga

I have now received all the referees reports on the above manuscript, and have reached a decision. The referees' comments are appended below, or you can access them online: please go to BenchPress and click on the 'Manuscripts with Decisions' queue in the Author Area.

The overall evaluation is positive and we would like to publish a revised manuscript in Development, provided that the referees' comments can be satisfactorily addressed. Please attend to all of the reviewers' comments in your revised manuscript and detail them in your point-by-point response. If you do not agree with any of their criticisms or suggestions explain clearly why this is so.

We are aware that you may currently be unable to access the lab to undertake experimental revisions. If it would be helpful, we encourage you to contact us to discuss your revision in greater detail. Please send us a point-by-point response indicating where you are able to address concerns raised (either experimentally or by changes to the text) and where you will not be able to do so within the normal timeframe of a revision. We will then provide further guidance. Please also note that we are happy to extend revision timeframes as necessary.

Reviewer 1

Advance summary and potential significance to field

The manuscript by Gamart and colleagues uses a lovely mix of careful embryology, molecular biology and bioinformatics to assess the function of Smad4 during limb bud development. In it, they

© 2021. Published by The Company of Biologists under the terms of the Creative Commons Attribution License (https://creativecommons.org/licenses/by/4.0/).

endogenously Flag tag the mouse Samd4 gene and use Chip-seq to identify binding sites. Then, making use of the conditional knockout allele of Smad4, in conjunction with a PrxCre, identify those genes who's expression changes in the absence of Smad4 within the limb-bud mesenchyme. Combining these data, with ATAC seq, they identify differentially expressed (both up and down regulated) SMAD4 target genes. These genes include members of the cholesterol synthesis pathway, which are reduced in expression and biochemical analysis shows that intracellular cholesterol is indeed reduced in the mutant. While Smad4 targets are identified in both the TGFB and BMP pathways, the manuscript shows that SMAD4 deficiency preferentially affects BMP signal transduction. In situ analysis highlights changes in the spatial expression patterns, predominantly in the anterior forelimb mesenchyme, and LacZ reporter analysis tests likely associated enhancers. The role of Smad4 in upregulating gene expression in the anterior mesenchyme is extended to identify genes which are also differentially expressed in the absence of Sonic hedgehog. The majority of these genes show discordant expression in the absence of either Smad4 or Shh. When the expression of the DEG is examined, it become apparent that anteriorly expressed genes are positively regulated by Smad4 and negatively regulated by Shh, while posteriorly expressed genes are oppositely regulated. This highlights unexpected opposing effects on co-regulated gene expression, in the case of Smad4 many of these being direct effects on target genes.

Comments for the author

I have only a few minor points I'd like the authors to clarify/ amend:
Somewhere on p10, it would be worth reminding the reader that while at these early stages the mutant limb bud is 'normal'- loss of Smad4 subsequently has catastrophic effects on limb bud development.
p14, 5 lines into the 2nd para- Should it read DEGs and targets – rather than E10 and E10.5 in the brackets?
Last line p16- include some comment on the expression of the CRM for Prrx2
Can figure 2 and 3, please be made to look the same? There are overlap lines in 3B but not 2B- the C panels and flipped relative to each other and the 'ns' in 3C is upside down. Also, I'm not sure the number of upregulated targets is right in 3D- they don't add up to 225- maybe 90 not 63?
In the heat maps of differential gene expression, comparing each individual sample to the average of the wildtypes makes sense. But in Figure 5 , where both expression at E10 and E10.5 is included, I think what they've done is compare everything to the average at E10, But they don't discuss this anywhere- please could they clarify?
There's a discrepancy between the panels in Figure 6 and the legend- it starts out ok then goes wrong by D.

<u>Reviewer 2</u>

Advance summary and potential significance to field

The manuscript describes the generation of a knock-in tagged smad4 allele and its use to determine the SMAD4 binding profile in the genome during limb development. This is combined with transcriptomic and in situ analysis of the expression of genes differentially expressed between smad4 mutant and control limbs. The findings support the idea of a predominant dedication of smad4 to transduce BMP signalling and demonstrates the direct and antagonic control by smad4 of a wide repertoir of common targets with the Shh pathway. This affects not only AP patterning genes but also PD patterning genes. In addition, the study uncovers a previously undescribed function of the BMP-Smad4 pathway in activating cholesterol synthesis enzymes in the limb bud. The tool generated 8smad4 knock-in) and the new pathways described are novel and will be of great interest for the limb model , but also for scientists interested in cholesterol synthesis regulation and, more generally, in BMP/TGFb signalling

Comments for the author

The manuscript reports very high quality and robust data identfying smad4 target genes and their cross-regulatory interaction with the Shh pathway. I only have minor questions to be addressed by the authors:

© 2021. Published by The Company of Biologists under the terms of the Creative Commons Attribution License (https://creativecommons.org/licenses/by/4.0/).

1.- Abstract: In the last sentence, the authors state: "the anterior/proximal and proximo/distal". I think they meant anterior/posterior instead of anteriro/proximal. In addition, "proximo/distal" appears here for the first time in the abstract, without any previous mention to the findings along this patterning axis, which is somehow strange.
2.- Pg 11: when assigning smad4 direct target genes, the authors use a a criterion that the transcriptional start should be positioned within 1 megabase of a SMAD4 binding site. In parallel, they show a strong correlation between the number of SMAD4 sits in a TAD and the probability thatt genes in that TAD respond to the elimination of smad4. Given that the ability of enhancers to regulate promoters is more dependent on their by colocatization in the same TAD that on the physical distance between them, would it not be more accurate to use colocalization in the same TAD as a criterion for selection of direct target genes?
3.- Pg 11. Again, on the criteria for target selection, while incorporating the criterion of an ATAC-seq open-chromatin status for the selection of genes activated by Smad4 seems convenient, for those targets repressed by smad4, it could be that real targets are missed because they are completely silent in the presence of smad4 and therefore do not show an ATAC-seq signal.
4.- Pg 15 and elsewhere in the manuscript. Regarding the specificity of Smad4 to BMP signalling, I see that at least some TGFb-specific targets seem to be bona-fide Smad4 targets. Even though BMP transduction seems predominant, I would suggest not to be so strong about discarding some relevance of smad4 in transducing tgfb signals in the limb bud.

First revision

Author response to reviewers' comments

Response to the comments and suggestions by the reviewers
We wish to thank both reviewers for their evaluation of our manuscript and positive recommendation concerning its suitability for publication. For convenience, we include a marked copy where changes are indicated in red.

The Editor requested that the revised manuscript text complies with Development's formatting guidelines in addition to addressing all comments of the two referees. As a result, we have shortened the abstract to less than 180 words, and the main text and figure legends from 7410 to 6877 words to be less than 7000 words. In addition, all information in figure legends was reformatted to fit the Development guidelines on replication and reproducibility.

Reviewer 1

Somewhere on p10, it would be worth reminding the reader that while at these early stages the mutant limb bud is 'normal'- loss of Smad4 subsequently has catastrophic effects on limb bud development.
We now describe this fact on page 8.

p14, 5 lines into the 2nd para- Should it read DEGs and targets - rather than E10 and E10.5 in the brackets?
Thank you for pointing this out- we have corrected the text accordingly (page 12)

Last line p16- include some comment on the expression of the CRM for Prrx2
We now describe the activity of the *Prrx2*-associated CRM in the result section on bottom of page 14 of the revised manuscript.

Can figure 2 and 3, please be made to look the same? There are overlap lines in 3B but not 2B- the C panels and flipped relative to each other and the 'ns' in 3C is upside down.
We added the missing overlap lines in 2B and rearranged the panels in 3C so that the order is the same in Fig. 2C and 3C.

Also, I'm not sure the number of upregulated targets is right in 3D- they don't add up to 225- maybe 90 not 63?

© 2021. Published by The Company of Biologists under the terms of the Creative Commons Attribution License (https://creativecommons.org/licenses/by/4.0/).

We are grateful to this reviewer for spotting the discrepancy. The number of up-regulated SMAD4 targets at E10.5 is 89 and not 63 (Table S7). When we initially performed the GO enrichment analysis for the up-regulated genes (upper panel of Fig. 3D), the list was accidently truncated. We have now repeated the entire GO analysis using the correct lists of up-regulated genes (and down-regulated genes). Most importantly, all conclusions from the GO analysis for both stages remain the same.

In the heat maps of differential gene expression, comparing each individual sample to the average of the wildtypes makes sense. But in Figure 5, where both expression at E10 and E10.5 is included, I think what they've done is compare everything to the average at E10, But they don't discuss this anywhere - please could they clarify?
This reviewer is correct, all samples are compared to the average at WT E10.0 so that the temporal changes in DEG expression can be compared between both stages for WT and *Smad4*$^{\Delta/\Delta c}$ forelimb buds. We now describe this in detail in the legend to Fig. 5 (page 50).

There's a discrepancy between the panels in Figure 6 and the legend- it starts out ok then goes wrong by D.
The legend of Fig. 6 has been amended to fit the Figure panels (page 51).

We thank the reviewer for spotting these mistakes that escaped the scrutiny of the authors.

Reviewer 2

1.- Abstract: In the last sentence, the authors state: "the anterior/proximal and proximo/distal". I think they meant anterior/posterior instead of anteriro/proximal. In addition, "proximo/distal" appears here for the first time in the abstract, without any previous mention to the findings along this patterning axis, which is somehow strange.
We agree and have removed the reference to "anterior/proximal" and "proximal/distal". In addition, we have shortened the abstract to the maximal length permitted by Development

2.- Pg 11: when assigning smad4 direct target genes, the authors use a criterion that the transcriptional start should be positioned within 1 megabase of a SMAD4 binding site. In parallel, they show a strong correlation between the number of SMAD4 sits in a TAD and the probability that genes in that TAD respond to the elimination of smad4. Given that the ability of enhancers to regulate promoters is more dependent on their by colocalization in the same TAD that on the physical distance between them, would it not be more accurate to use colocalization in the same TAD as a criterion for selection of direct target genes?
We initially used a 1Mb interval as an informed choice because this is around the size of an average TAD (Dixon et al. 2016, https://doi.org/10.1016/j.molcel.2016.05.018). We now describe this criterion on page 8. In addition, we also analysed the potential regulatory activities of each predicted binding site based on TADs (Fig. 2C and Fig 3C), in line with the same rationale suggested by the reviewer. We also explicitly added the 1Mb maximal physical distance as an additional criterion, given that not all genes in the genome are predicted to be contained in a TAD.

3.- Pg 11. Again, on the criteria for target selection, while incorporating the criterion of an ATAC-seq open-chromatin status for the selection of genes activated by Smad4 seems convenient, for those targets repressed by smad4, it could be that real targets are missed because they are completely silent in the presence of smad4 and therefore do not show an ATAC-seq signal.
The rationale for this is that most transcription factors (TF) bind accessible DNA when they either act as transcriptional activators or repressors. Overlapping ChIP-seq and ATAC-seq is a widely used approach to increase the specificity and sensitivity of the binding events predicted by ChIP-seq alone. More details on the relevance of using ATAC-seq in combination with ChIP-seq analysis to identify the significant interactions of TFs with DNA can be found in a review by Yan et al. 2020 (https://doi.org/10.1186/s13059-020-1929-3).

4.- Pg 15 and elsewhere in the manuscript. Regarding the specificity of Smad4 to BMP signalling, I see that at least some TGFb-specific targets seem to be bona-fide Smad4 targets. Even though BMP transduction seems predominant, I would suggest not to be so strong about discarding some

© 2021. Published by The Company of Biologists under the terms of the Creative Commons Attribution License (https://creativecommons.org/licenses/by/4.0/).

relevance of smad4 in transducing tgfb signals in the limb bud.
We agree with the reviewer that TGFß signaling could play a role even if convincing genetic evidence is lacking and in fact, we cite two papers (Karamboulas et al. page 4 and page 18; and Pelikan et al. page 20) that refer to TGFß activity. We have altered the text accordingly to better reflect this fact.
Page 13: "As no corresponding changes are detected in the TGFß pathway (Fig. 5D, Fig. S4), *Smad4* functions predominantly ~~or exclusively~~ in BMP signal transduction during early limb bud development (E10.0-E10.5).
Page 18: "While crosstalk between BMP and TGFß SMAD4-mediated signal transduction is *possible* (Karamboulas et al., 2010), SMAD4 is *specifically* required for BMP-signal transduction in early limb buds as the expression of *Bmp* ligands is up-regulated in *Smad4*-deficient limb buds…" is changed into: "Crosstalk between BMP and TGFß SMAD4-mediated signal transduction is **likely** (Karamboulas et al., 2010), however our study suggests that SMAD4 is **predominantly** required for BMP-signal transduction in early limb buds as the expression of *Bmp* ligands is up-regulated in *Smad4*-deficient limb buds…"

Second decision letter

MS ID#: DEVELOP/2021/200182

MS TITLE: SMAD4 target genes are part of a transcriptional network that integrates the response to BMP and SHH signaling during early limb bud patterning

AUTHORS: Julie Gamart, Iros Barozzi, Frédéric Laurent, Robert Reinhardt, Laurène Ramos Martins, Thomas Oberholzer, Axel Visel, Rolf Zeller, and Aimée Zuniga
ARTICLE TYPE: Research Article

I am happy to tell you that your manuscript has been accepted for publication in Development, pending our standard ethics checks.

Reviewer 1

Advance summary and potential significance to field

I'm happy with the revisions made to the manuscript.

Comments for the author

I'm happy with the revisions made to the manuscript.

© 2021. Published by The Company of Biologists under the terms of the Creative Commons Attribution License (https://creativecommons.org/licenses/by/4.0/).
